# Mapping the venture capital and private equity research: a bibliometric review and future research agenda

**DOI:** 10.1007/s11187-022-00684-9

**Published:** 2022-10-04

**Authors:** Douglas Cumming, Satish Kumar, Weng Marc Lim, Nitesh Pandey

**Affiliations:** 1 College of Business, Florida Atlantic University, 777 Glades Road, Boca Raton, FL 33431 USA; 2 Birmingham Business School, Universty of Birmingham, University House, 116 Edgbaston Park Rd, Birmingham, B15 2TY UK; 3 Malaviya National Institute of Technology, Jaipur, India; 4 School of Business, Swinburne University of Technology, Jalan Simpang Tiga, 93350 Kuching, Sarawak Malaysia; 5 Swinburne Business School, Swinburne University of Technology, John Street, Hawthorn, Victoria 3122 Australia

**Keywords:** Bibliometric analysis, Venture capital, Private equity, G30, G32

## Abstract

The fields of venture capital and private equity are rooted in financing research on capital budgeting and initial public offering (IPO). Both fields have grown considerably in recent times with a heterogenous set of themes being explored. This review presents an analysis of research in both fields. Using a large corpus from the Web of Science, this study used bibliometric analysis to present a comprehensive encapsulation of the fields’ geographical focus, methodological choices, prominent themes, and future research directions. Noteworthily, the foundational themes in venture capital research are *venture capital adoption and financing processes*, *venture capital roles in business*, *venture capital governance*, *venture capital syndication*, and *venture capital and creation of public organizations.* In private equity research, *style drift into venture capital* emerges as a key theme alongside *buyouts and privatization*, and *valuation and performance of private equity investment*.

## Abstract (plain language summary)

Financing is an important aspect of business that creates opportunities for investors and invested entities. Venture capital and private equity are two major vehicles of financing a business. While venture capital manifests as small investments that support the business operations of a large number of promising firms (more risky) in the early stage (startup), private equity transpires as large investments that support the business expansion of a small number of stable firms (less risky) in the later stage (scaleup). This study presents state-of-the-art insights into the current trends and future directions of venture capital and private equity using a bibliometric analysis of high-quality research on these forms of financing.

## Introduction

Venture capital is one of the primary methods by which private equity investors make funds available to startups, early-stage, and emerging companies that have high growth potential. Arising out of financing research on innovation and economic growth (Hsu & Kenney, 2005), venture capital is a widely researched area among entrepreneurship and finance scholars. Similarly, private equity, which hosts venture capital, has also seen considerable growth in its research and practice. Investors usually contribute to private equity funds to capitalize on investment opportunities that may not be available to them through other channels of investment (Fuchs et al., 2021, 2022). Private equity is often considered costlier than public equity (Brav, 2009), causing private firms to choose debt financing. However, with the deregulation of capital markets, large investors’ access to private equity funds has increased (Ewens & Farre-Mensa, 2020), which has potentially increased their ability to fund businesses, thus leading to a decline in initial public offering (IPO). The assets managed by PE firms have increased by nearly 70% in the past five years (Dai, 2022). Today, venture capitalists and private equity funders engage in a variety of activities, including socially responsible investing or impact investing (Barber et al., 2021), with venture capital being seen as one of the more prominent methods of financing new ventures (Ho & Wong, 2007) along with being signal for the quality of the venture for outside investors (Revest & Sapio, 2012).

Many studies on venture capital and private equity exist, and they continue to proliferate over time. This may be attributed to the increased prominence and role of venture capital and private equity funds in the capital market. A closer look at such studies in this review reveals that research on venture capital and private equity is rooted in capital budgeting and IPO research, and early studies in the field have studied venture capital and private equity from that perspective. With the increasing role of venture capital and private equity funds and their diversified portfolios, the two fields have grown in authority with various prominent subfields, and thus, they are investigated separately in recognition of venture capital as a substantially large field of research that warrants its own scrutiny and private equity as a core and mature field of research. With the growth of venture capital and private equity research and the increasing heterogeneity of topics investigated, there is a need to conduct a comprehensive review of studies in both fields in order to take stock of their performance and scientific contributions. Noteworthily, a field can only advance when new research extends prior research, and crucial to that endeavor is a good understanding of the state of the field.

To this end, this study aims to present a comprehensive encapsulation of venture capital and private equity research. Two separate datasets of literature corpus—i.e., venture capital and private equity—are sourced and scrutinized to gain insights into the performance and science of research in both fields. For this purpose, this study takes up several objectives, which are further refined into research questions.

The first objective of this study is to present a performance analysis of venture capital and private equity research, including the fields’ primary contributors. A performance analysis is quite common among literature review studies (Donthu et al., 2021). The analysis of this style may seem overly descriptive to veterans of both fields, but it is invaluable to emerging scholars—particularly the ones pursuing their PhDs. Specifically, the analysis presents new scholars with knowledge of where to look for quality research in both fields. Yet, veterans may consider such insights positively too when they choose to view it as an opportunity to gain an objective and updated overview of the progress of both fields at a glance without engaging in duplicative efforts to gain the same insight. Moreover, they stand to gain recognition for their contributions in terms of productivity and impact, as this study will reveal. Based on the discussion, we present the following research questions:

- **RQ1.** What are the publication patterns in the fields of venture capital and private equity?
- **RQ2.** Who are the most prolific contributors to the fields of venture capital and private equity?
- **RQ3.** Which are the most cited articles in the fields of venture capital and private equity?

The second objective of this study is to present an analysis of the most dominant methodologies in venture capital and private equity research, including the classification of research in both fields across the different research approaches, designs, and data types (Baker et al., 2020). In addition, this study will also present the geographical regions in which venture capital and private equity studies have predominantly taken place. The classification of geographical regions in this study is distinct from the typical list of countries that are most prolific in publishing research in the field; instead, the classification herein will focus on the *source* of the samples for each study. This is particularly insightful in today’s world of financial research, where an author from the USA can conduct research with sample data from an Asian country—and vice versa. The benefits of this exploration are twofold. First, it demonstrates where the field stands in terms of both geographical and methodological concentration, potentially identifying gaps in the literature for future research to address. Second, new scholars will find it helpful to discover the dominant methodologies and their temporal trends, as this will equip them with knowledge of which methodologies they may utilize in their future research. This, in turn, will help new scholars find their footing in the field. Consequently, we present the following research questions:

- **RQ4.** On which geographical regions have scholars focused in the fields of venture capital and private equity research, and which geographical regions have scholars ignored?
- **RQ5.** What are the dominant methodologies in the fields of venture capital and private equity research?

The third and final objective of this study is to present a science mapping (Cobo et al., 2011; Donthu et al., 2021) of venture capital and private equity research, including the analysis of collaboration patterns, research themes, and trends. The study of collaboration in a field can be extremely helpful in understanding its research (Crane, 1969) because the social structures created by collaborations are important to the field’s development. For example, group A may be pursuing a different subarea of venture capital or private equity research than group B. It is then interesting to analyze how both groups interact with one another, as well as which group is more dominant and prolific in the field. Indeed, this analysis indicates the emergence, decline, and interaction between different subareas of research. Apart from collaboration patterns, this study also focuses on thematic analysis, which is perhaps the most important part of any review because it focuses on the content of the studies themselves. By finding different thematic clusters—in both the entire field and the research published more recently (Andersen, 2019)—this study will provide the foundational themes in the fields’ research, their development over time, and propositions for future research. We thus present the final two research questions:

- **RQ6.** What are the collaboration patterns in the fields of venture capital and private equity research?
- **RQ7.** What are the foundational themes in the fields of venture capital and private equity research, and what are the ways forward for the fields?

The rest of the article is organized as follows. Section 2 presents an overview of the bibliometric methodology. Subsequently, Sect. 3 presents the results of the performance analysis of venture capital and private equity research using the above-mentioned research questions. The thematic analysis for venture capital and private equity is conducted in Sects. 4 and 5, respectively. Finally, we conclude the study in Sect. 6.

## Methodology

To delve more deeply into the growing literature on venture capital and private equity research, this study combined systematic literature review (SLR) (Tranfield et al., 2003) and bibliometric analysis (Donthu et al., 2021). The former introduces a method of review that is transparent, replicable, and more authentic. However, the qualitative nature of SLR may be a drawback because qualitative reviews often suffer from interpretation bias (MacCoun, 1998). Interpretation bias, for its part, implies that the interpretation of any work is dependent upon a given scholar’s background. Another drawback is that large works cannot be reviewed qualitatively. We used bibliometric analysis to present a solution to such drawbacks. First, the quantitative nature of bibliometric analysis can help minimize interpretation bias. Second, bibliometric analysis can be used with large works (Ramos-Rodrígue & Ruíz-Navarro, 2004). We follow the guidelines of Mukherjee et al., 2022 in applying bibliometric method. Table 1 presents the mapping of the research objectives and the tools that we used to achieve them.

**Table 1:** Mapping of research objectives to methodology

Research objectives	Analytical strategy	Analytical tool	Data type	Technology
• To present a performance analysis of venture capital and private equity research	Delineate publication and citation patterns of contributors and contributions	Performance analysis	• Publications • Citations • *h*-index	Database: • Web of Science Software: • VOSviewer • Gephi
• To present an analysis of the most dominant methodologies and geographical focuses in venture capital and private equity research	Identify methodological and geographical trends of publications	Descriptive analysis	• Full text (methodology, sample country)	
• To present a science mapping of venture capital and private equity research	Corroborate different content markers and develop thematic clusters	• Co-authorship analysis • Co-citation analysis • Bibliographic coupling	• Authors • References	

Using multiple rounds of filtering, we used the SLR methodology to find potentially relevant literature from the keyword search. For both venture capital and private equity, we used the Web of Science database. After the keyword search (i.e., “private equity” for private equity research and “venture capital” for venture capital research), we found several studies in both areas. In order to enable a focused review of the state of research in both fields, the publications appearing in the private equity dataset were removed from venture capital dataset. Subsequently, we refined the results to the relevant Web of Science categories, such as business finance, business, management, and economics, resulting in 1550 documents for the venture capital corpus and 941 documents for the private equity corpus.

Since the article search was conducted on the Web of Science, an article’s inclusion or exclusion is subject to two main conditions. *First*, the article must be published after 2000 and should be a part of the Web of Science Core Collection. *Second*, the article must have either “private equity” (for inclusion in private equity dataset) or “venture capital” (for inclusion in venture capital dataset) in at least one of four fields: “title,” “abstract,” “author keywords,” and “KeywordPlus” (i.e., keywords assigned by the Web of Science). Bibliometric studies such as the present study typically rely on bibliographic data from the scientific database, and thus, any errors in the database could affect the dataset for the study. Despite the potential of errors, the impact of such errors is likely to be negligible because (1) the authors did a follow-up to carefully check and correct for recognizable errors (e.g., missing data—e.g., author name), and (2) the corpus for the study is large enough for major themes to emerge. In the Appendix, we explain the choice of the sample period from 2001 to 2021.

We then conducted an analysis of the literature using a range of bibliometric analysis tools to achieve our research objectives. To conduct a performance analysis of the field, we used citations and publications as measures of influence and productivity (Ding et al., 2009).

For our second research objective, the articles were classified on the basis of their research approach (i.e., empirical, conceptual, modeling and analytical, review, or mixed) and design (i.e., quantitative, qualitative, or mixed) (Baker et al., 2020). We further classified the articles according to the source of their sample (i.e., archival, survey, case study, interview, experimental, or field).

For our final research objective, we used investigative tools such as co-authorship analysis (Acedo et al., 2006), co-citation analysis (Hota et al., 2019; Samiee & Chabowski, 2012; Xu et al., 2018), and bibliographic coupling (Baker et al., 2020). The large size of the literature required us to find content markers. In the case of co-authorship, the content markers were the authors themselves. However, in the case of co-citation analysis and bibliographic coupling, the content markers were references. Notably, for co-citation analysis, articles share a thematic similarity when they are frequently cited together (Small, 1973). The study of such works using co-citation is instrumental in identifying the development of paradigms in a subject field. The development of paradigms is further an indication of ideological consensus among scholars (Culnan et al., 1990). Thus, the study of cited references is instrumental in understanding the themes which are widely upon by scholars. For bibliographic coupling, articles generally share literature references (Weinberg, 1974). These thematic similarities were then used to create clusters of articles and determine the key themes of research in both fields.

For the purpose of conducting co-authorship analysis, co-citation analysis, and bibliographic coupling, we used different software packages, including VOSviewer (van Eck & Waltman, 2010) for science mapping and Gephi (Bastian et al., 2009) for network visualization.

## Performance of analysis of venture capital and private equity research

To achieve our objectives and conduct a performance analysis of the field, we presented three research questions. The answers to these questions, in turn, revealed which subareas of the field have grown and which have not. Our first research question deals with publication patterns in the field of venture capital and private equity research. To achieve our first research objective, we conducted a performance analysis of venture capital and private equity research.

**Fig. 1:**
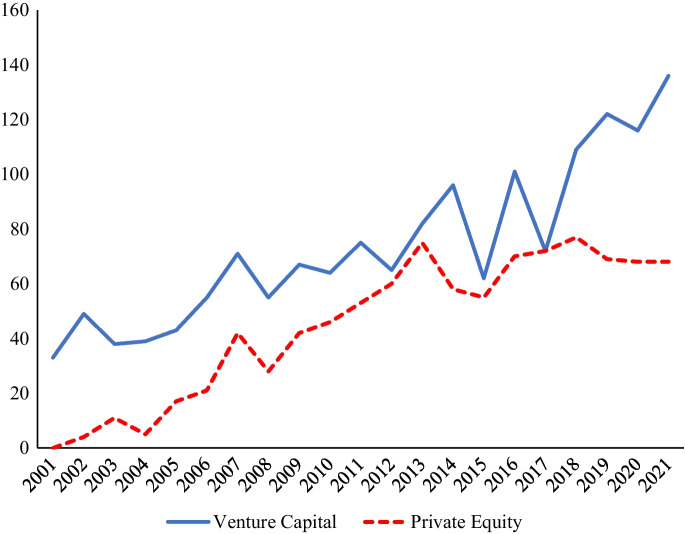
Year-wise publications for venture capital and private equity research. *Note:* Publications included in 2021 are available in WoS up to October 2021

The solid line in Fig. 1 presents the publication trend for venture capital research. The publication trend (RQ1) suggests that the field’s research has grown organically over the years. In other words, research in the field does not seem to be spurred by any externality or event, with more than 30 publications each year. Whereas, the dotted line in Fig. 1 shows that public equity research has grown consistently since 2001. The growth here is evident; since 2012, on average, more than 60 private equity studies have been published. Interestingly, more private equity studies appear to have been published after 2008. The focus on alternative sources of financing (other than IPOs or bank loans) may have influenced this increased interest in private equity research subsequent to 2008, which was the year that the global financial crisis affected many firms.

**Fig. 2:**
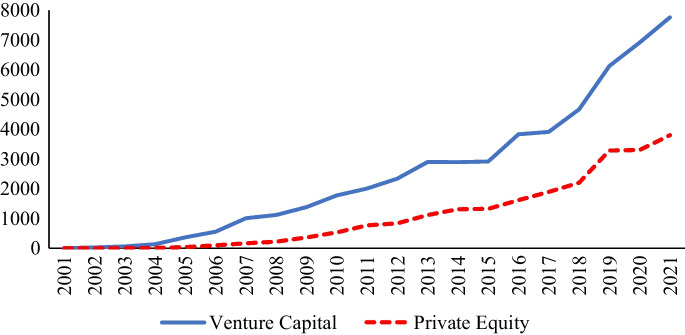
Year-wise citations for venture capital and private equity research. *Note*: Citations are based on publications in WoS up to October 2021

Figure 2 shows the trend of citations for both venture capital and private equity research in the review corpus. The citations for both fields were zero in 2001, which is expected since the dataset begins in that year. Nevertheless, the citations for both fields have grown over the years, with venture capital research achieving almost 8000 citations while private equity research receiving nearly 4000 citations in 2021.

Table 2 presents the list of the most prolific authors for venture capital and private equity research (RQ2). In the case of venture capital research, the most prolific researcher in the field is Douglas Cumming, who has 28 publications, followed by Mike Wright with 25 publications and Colin Mason with 17 publications. Mike Wright is also the most impactful author who has attracted 1823 citations for his research on venture capital, followed by Thomas Hellmann with 1594 citations and Douglas Cumming with 1,531 citations. In the case of private equity research, Mike Wright emerges as the most prolific author, with 50 publications, followed by Douglas Cumming, who has 47 publications and Sofia Johan with 20 publications. In terms of citations, Douglas Cumming is the leader (2271), followed by Mike Wright (1563) and Steven A. Kaplan (1407).

**Table 2:** Most prolific authors

Author	Latest/last reported affiliation	Publications	Citations	*h*-index
**Panel A: Most prolific authors for venture capital research**				
Douglas Cumming	Florida Atlantic University, USA	28	1,531	20
Mike Wright	Imperial College London, UK	25	1,823	19
Colin M. Mason	University of Glasgow, UK	17	1,019	12
Markku V.J. Maula	Aalto University, Finland	15	882	14
Richard T. Harrison	University of Edinburgh, UK	15	655	10
Armin Schwienbacher	SKEMA Business School, France	13	516	10
Andy Lockett	University of Warwick, UK	12	1,199	12
Sophie Manigart	Vlerick Business School, Belgium	12	615	11
Massimo G. Colombo	Politecnico di Milano, Italy	10	563	8
Tom Vanacker	Ghent University, Belgium	10	260	8
Thomas Hellmann	University of Oxford, UK	9	1,594	8
Josh Lerner	Harvard Business School, USA	9	1,287	8
Christian Keuschnigg	University of Innsbruck, Austria	9	664	9
Haemin Dennis Park	University of Texas at Dallas, USA	9	185	5
Gary Dushnitsky	London Business School, UK	8	1,056	8
Dean A. Shepherd	Indiana University, USA	8	855	8
Dimo Dimov	University of Bath, UK	8	659	8
Luca Grilli	Politecnico di Milano, Italy	8	481	6
Yong Li	University of Nevada, Las Vegas, USA	8	472	8
Jarunee Wonglimpiyarat	Thammasat University, Thailand	8	126	6
**Panel B: Most prolific authors for private equity research**				
Mike Wright	Imperial College London, UK	50	1,563	21
Douglas Cumming	Florida Atlantic University, USA	47	2,271	28
Sofia Johan	Florida Atlantic University, USA	20	432	14
Josh Lerner	Harvard Business School, USA	16	1,364	14
Ludovic Phalippou	Said Business School, UK	13	520	10
Sophie Manigart	Vlerick Business School, Belgium	13	221	9
Berk A. Sensoy	Vanderbilt University, USA	12	302	9
Steven N. Kaplan	University of Chicago, USA	11	1,407	9
Igor Filatotchev	King's College London	11	549	9
Geoffrey Wood	Western University, Canada	11	185	7
Silvio Vismara	University of Bergamo, Italy	10	377	9
Annalisa Croce	Politecnico di Milano, Italy	10	313	8
Armin Schwienbacher	SKEMA Business School, France	10	185	8
Michael S. Weisbach	Ohio State University, USA	9	332	7
Tim Jenkinson	Oxford University, UK	9	297	4
Axel Buchner	University of Passau, Germany	9	55	5
Luca Grilli	Politecnico di Milano, Italy	8	344	7
Miguel Meuleman	Vlerick Business School, Belgium	8	302	7
Jose Marti	Universidad Complutense de Madrid, Spain	7	237	5
Fabio Bertoni	SKEMA Business School, France	7	230	6

Note: The figures presented in the table are based on the Web of Science Core collection between the period 2001 and 2021

Table 3 shows the most prolific sources for venture capital and private equity research (RQ2). In terms of venture capital research, *Journal of Business Venturing*, which hosts 85 publications, is the most prolific source for research in the field, followed by *Small Business Economics* with 69 publications. In terms of impact, the most impactful journal is *Journal of Finance*, which has amassed 2976 citations for research on venture capital, followed by *Journal of Business Venturing* with 2694 citations. In terms of private equity research, *Journal of Corporate Finance* has the most publications, followed by *Journal of Financial Economics*. Between 2001 and 2021, both journals published more than 50 articles each on private equity. Finally, in terms of citations calculated based on the citations received from within our dataset, *Journal of Finance* has been cited the greatest number of times (1611), followed by *Journal of Financial Economics* (1591) and *Review of Financial Studies* (584), respectively.

**Table 3:** Most prolific and impactful sources

Source	Publications	Source	Local citations
**Panel A: Most prolific and impactful sources for venture capital research**			
*Journal of Business Venturing*	85	*Journal of Finance*	2976
*Small Business Economics*	69	*Journal of Business Venturing*	2694
*Venture Capital*	64	*Journal of Financial Economics*	1868
*Research Policy*	44	*Strategic Management Journal*	969
*Journal of Corporate Finance*	43	*Administrative Science Quarterly*	888
*Entrepreneurship Theory and Practice*	41	*Research Policy*	492
*Strategic Entrepreneurship Journal*	31	*American Journal of Sociology*	488
*Journal of Financial Economics*	31	*Academy of Management Journal*	463
*Strategic Management Journal*	29	*Review of Financial Studies*	445
*Journal of Business Research*	26	*Management Science*	397
*International Journal of Technology Management*	25	*Financial Management*	380
*Journal of Technology Transfer*	24	*Review of Economic Studies*	379
*Academy of Management Journal*	23	*RAND Journal of Economics*	330
*Journal of Small Business Management*	23	*Journal of Corporate Finance*	321
*Technological Forecasting and Social Change*	22	*Entrepreneurship Theory and Practice*	297
*Journal of Banking and Finance*	21	*The Venture Capital Cycle*	288
*Forbes*	18	*Organization Science*	285
*International Entrepreneurship and Management Journal*	18	*Academy of Management Review*	272
*Review of Financial Studies*	17	*Journal of Banking and Finance*	269
*Organization Science*	17	*Quarterly Journal of Economics*	213
**Panel B: Most prolific and impactful sources for private equity research**			
*Journal of Corporate Finance*	58	*Journal of Finance*	1,611
*Journal of Financial Economics*	51	*Journal of Financial Economics*	1,591
*Small Business Economics*	34	*Review of Financial Studies*	584
*Forbes*	29	*Journal of Business Venturing*	517
*Journal of Banking and Finance*	28	*Journal of Corporate Finance*	421
*Review of Financial Studies*	27	*American Economic Review*	296
*Journal of Portfolio Management*	26	*Journal of Banking and Finance*	262
*European Financial Management*	26	*Quarterly Journal of Economics*	187
*Journal of Finance*	23	*Review of Economic Studies*	126
*Fortune*	21	*Entrepreneurship Theory and Practice*	107
*Journal of Business Venturing*	20	*RAND Journal of Economics*	90
*Venture Capital*	18	*Financial Management*	76
*Corporate Governance: An International Review*	15	*Review of Economics and Statistics*	75
*British Journal of Management*	14	*Econometrica*	75
*European Business Organization Law Review*	13	*Journal of Empirical Finance*	73
*Entrepreneurship Theory and Practice*	12	*Journal of Business*	62
*Harvard Business Review*	12	*Journal of Business Finance and Accounting*	61
*Strategic Management Journal*	11	*Journal of Public Economics*	52
*Journal of Financial and Quantitative Analysis*	11	*International Journal of Management Reviews*	50
*Journal of Business Ethics*	11	*Research Policy*	43

Local citations refer to citations received from publications within the dataset used in this study. The publication figures are based on the Web of Science Core collection covering a period between 2001 and 2021

Table 4 shows the list of the most cited articles on venture capital and private equity research (RQ3). In terms of venture capital research, the most cited article in the field is Lee et al., (2001) article on technology-based ventures, which has been cited 982 times. This is followed by Hellmann and Puri’s (2002) article on the role of venture capital in the professionalization of startups, which has been cited 798 times. The third most cited article is Pittaway et al.’s (2004) article on relationship between networking and innovation, wherein venture capital is discussed as a network partner having influence over innovation. This article has been cited 773 times. In terms of private equity research, Kaplan and Schoar’s (2005) study on private equity return is the most cited article, with 590 citations, followed by Moskowitz and Vissing-Jørgensen’s (2002) study on entrepreneurial finance (380 citations) and Kaplan and Strömberg’s (2009) study on leveraged buyouts (301 citations), respectively. The table also suggests that finance journals publish the most impactful studies in the field.

**Table 4:** Most cited articles

Author(s)	Title	Year	Journal	TC	C/Y
**Panel A: Most cited articles on venture capital since 2001**					
Lee, C; Lee, K; Pennings, JM	Internal capabilities, external networks, and performance: A study on technology-based ventures	2001	*Strategic Management Journal*	982	46.76
Hellmann, T; Puri, M	Venture capital and the professionalization of start-up firms: Empirical evidence	2002	*Journal of Finance*	798	39.90
Pittaway, L; Robertson, M; Munir, K; Denyer, D; Neely, A	Networking and innovation: A systematic review of the evidence	2004	*International Journal of Management Reviews*	773	42.94
Shane, S; Stuart, T	Organizational endowments and the performance of university start-ups	2002	*Management Science*	690	34.50
Kaplan, SN; Stromberg, P	Financial contracting theory meets the real world: An empirical analysis of venture capital contracts	2003	*Review of Economic Studies*	686	36.11
Hall, BH	The financing of research and development	2002	*Oxford Review of Economic Policy*	684	34.20
Hochberg, YV; Ljungqvist, A; Lu, Y	Whom you know matters: Venture capital networks and investment performance	2007	*Journal of Finance*	681	45.40
Di Gregorio, D; Shane, S	Why do some universities generate more start-ups than others?	2003	*Research Policy*	661	34.79
Gompers, P; Lerner, J	The venture capital revolution	2001	*Journal of Economic Perspectives*	600	28.57
Baum, JAC; Silverman, BS	Picking winners or building them? Alliance, intellectual, and human capital as selection criteria in venture financing and performance of biotechnology startups	2004	*Journal of Business Venturing*	594	33.00
Zucker, LG; Darby, MR; Armstrong, JS	Commercializing knowledge: University science, knowledge capture, and firm performance in biotechnology	2002	*Management Science*	527	26.35
Hsu, DH	What do entrepreneurs pay for venture capital affiliation?	2004	*Journal of Finance*	505	28.06
Gulati, R; Higgins, MC	Which ties matter when? The contingent effects of interorganizational partnerships on IPO success	2003	*Strategic Management Journal*	450	23.68
Kaplan, SN; Stromberg, P	Characteristics, contracts, and actions: Evidence from venture capitalist analyses	2004	*Journal of Finance*	395	21.94
Sorensen, M	How smart is smart money? A two-sided matching model of venture capital	2007	*Journal of Finance*	363	24.20
Hsu, DH	Experienced entrepreneurial founders, organizational capital, and venture capital funding	2007	*Research Policy*	315	21.00
Lee, PM; Wahal, S	Grandstanding, certification and the underpricing of venture capital backed IPOs	2004	*Journal of Financial Economics*	285	15.83
Lechner, C; Dowling, M; Welpe, I	Firm networks and firm development: The role of the relational mix	2006	*Journal of Business Venturing*	280	17.50
Wright, M; Lockett, A; Clarysse, B; Binks, M	University spin-out companies and venture capital	2006	*Research Policy*	279	17.44
Dushnitsky, G; Lenox, MJ	When do incumbents learn from entrepreneurial ventures? Corporate venture capital and investing firm innovation rates	2005	*Research Policy*	277	16.29
**Panel B: Most cited articles on private equity since 2001**					
Kaplan, SN; Schoar, A	Private equity performance: Returns, persistence, and capital flows	2005	*Journal of Finance*	590	34.71
Moskowitz, TJ; Vissing-Jorgensen, A	The returns to entrepreneurial investment: A private equity premium puzzle?	2002	*American Economic Review*	380	19.00
Kaplan, SN; Stromberg, P	Leveraged buyouts and private equity	2009	*Journal of Economic Perspectives*	301	23.15
Lerner, J; Schoar, A	Does legal enforcement affect financial transactions? The contractual channel in private equity	2005	*Quarterly Journal of Economics*	219	12.88
Phalippou, L; Gottschalg, O	The performance of private equity funds	2009	*Review of Financial Studies*	209	16.08
Bruton, GD; Filatotchev, I; Chahine, S; Wright, M	Governance, ownership structure, and performance of IPO firms: The impact of different types of private equity investors and institutional environments	2010	*Strategic Management Journal*	200	16.67
Denis, DJ	Entrepreneurial finance: An overview of the issues and evidence	2004	*Journal of Corporate Finance*	199	11.06
Metrick, A; Yasuda, A	The economics of private equity funds	2010	*Review of Financial Studies*	181	15.08
Brav, O	Access to capital, capital structure, and the funding of the firm	2009	*Journal of Finance*	180	13.85
Cumming, D; Walz, U	Private equity returns and disclosure around the world	2010	*Journal of International Business Studies*	179	14.92
Cumming, D; Siegel, DS; Wright, M	Private equity, leveraged buyouts and governance	2007	*Journal of Corporate Finance*	160	10.67
Bargeron, LL; Schlingemann, FP; Stulz, RM; Zutter, CJ	Why do private acquirers pay so little compared to public acquirers?	2008	*Journal of Financial Economics*	134	9.57
Cumming, D	Government policy towards entrepreneurial finance: Innovation investment funds	2007	*Journal of Business Venturing*	129	8.60
Lerner, J; Schoar, A; Wongsunwai, W	Smart institutions, foolish choices: The limited partner performance puzzle	2007	*Journal of Finance*	127	8.47
Badertscher, BA; Katz, SP; Rego, SO	The separation of ownership and control and corporate tax avoidance	2013	*Journal of Accounting and Economics*	113	12.56
Givoly, D; Hayn, CK; Katz, SP	Does public ownership of equity improve earnings quality?	2010	*Accounting Review*	110	9.17
Stuart, TE; Yim, S	Board interlocks and the propensity to be targeted in private equity transactions	2010	*Journal of Financial Economics*	101	8.42
Meuleman, M; Amess, K; Wright, M; Scholes, L	Agency, strategic entrepreneurship, and the performance of private equity-backed buyouts	2009	*Entrepreneurship Theory and Practice*	98	7.54
Renneboog, L; Simons, T; Wright, M	Why do public firms go private in the UK? The impact of private equity investors, incentive realignment and undervaluation	2007	*Journal of Corporate Finance*	97	6.47
Groh, AP; von Liechtenstein, H; Lieser, K	The European venture capital and private equity country attractiveness indices	2010	*Journal of Corporate Finance*	94	7.83

*TC* total citations according to the Web of Science. *C/Y* citations per year. The numbers presented in the table are based on the Web of Science Core Collection for period between 2001 and 2021, and the articles are listed based on their total citations

**Fig. 3:**
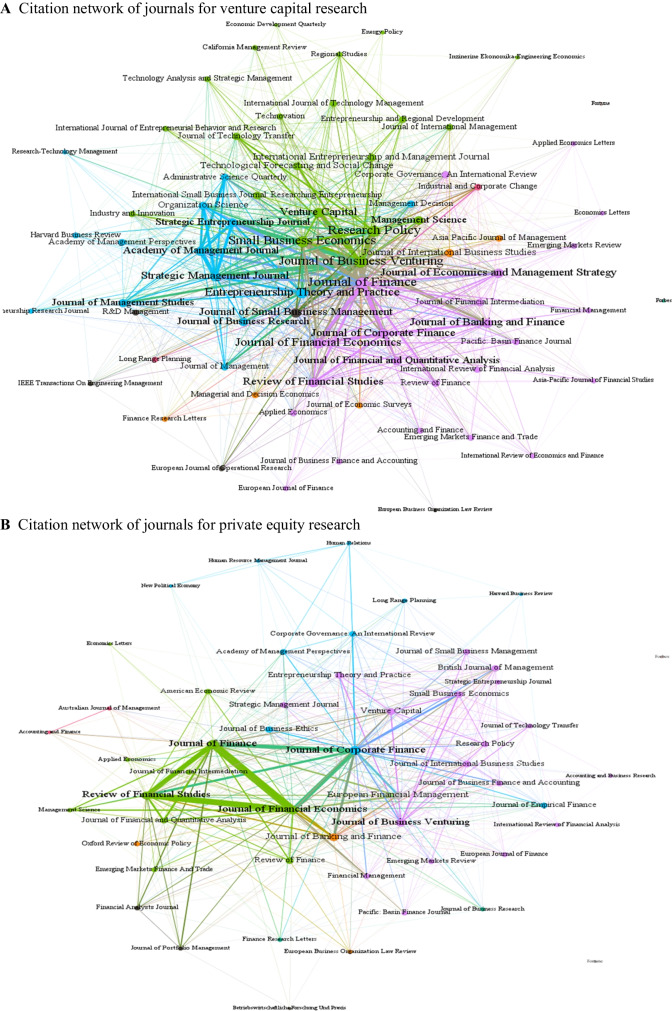
Citation network of journals for venture capital and private equity research. **A** Citation network of journals for venture capital research. **B** Citation network of journals for private equity research

Figure 3 shows the citation network for journals hosting venture capital and private equity research. In the case of venture capital research, we find a denser network, with journals showing a much closer connectivity with one another. One striking feature in this network is the strong citation bonds shared by finance (e.g., *Journal of Finance*), management (e.g., *Organization Science*, *Strategic Management Journal*), and entrepreneurship (arguably a subset of management journals—e.g., *Research Policy*, *Journal of Business Venturing*) journals. The clusters in this network are largely representative of the area. The ones in purple in this network are finance journals, whereas the ones in blue and green are management and entrepreneurship journals, respectively. However, the network for private equity research is quite different. In this network, the journals from the same area do not share strong bonds. The finance journals, which are dominant in this field, share strong citation links amongst themselves. The “top three” finance journals—i.e., *Journal of Finance*, *Journal of Financial Economics*, and *Review of Financial Studies*—cite one another quite often, and less often than other journals. *Journal of Corporate Finance* is prominent in this network, citing the “top three” finance journals quite often. In this network, we did not find many strong links between finance and non-finance journals, which is consistent with Cumming and Johan (2017). Noteworthily, the citations of private equity research are highly focused on finance, which contrasts against the management and entrepreneurship citation patterns witnessed in venture capital research, where management and entrepreneurship journals share strong citation bonds, albeit not nearly as strong as the citations for journals within their own discipline.

**Fig. 4:**
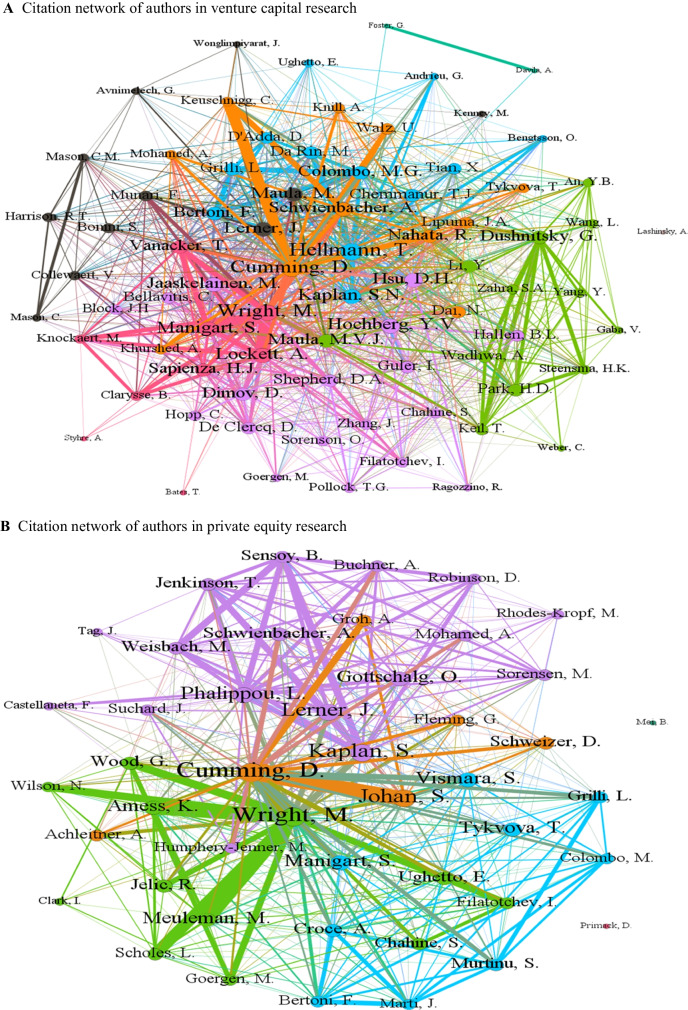
Citation network of authors in venture capital and private equity research. **A** Citation network of authors in venture capital research. **B** Citation network of authors in private equity research

Figure 4 shows the citation network of authors publishing on venture capital and private equity. In this case, the networks for both fields are quite similar, where it is observable that authors who work together also have strong citation ties. In the case of venture capital research, examples include Mike Wright, Andy Lockett, Sophie Manigart, Harry Sapienza, and Mirjam Knockaert, whereas, in the case of private equity research, examples include Steven Kaplan, Berk Sensoy, Tim Jenkinson, David Robinson, and Michael Weisbach. This observation could be explained by the possibility that authors form citations links with co-authors from their research group as well as current and former Ph.D. students. Cumming and Johan (2017) discuss a variety of other behaviors that may drive citation patterns in the literature and the choices authors make to submit their work to finance versus management and entrepreneurship journals.

## Thematic analysis of venture capital research

### Geographical focus and methodological choice analysis

To achieve our second research objective, we conducted a geographical focus and methodological choice analysis. Table 5 shows a summary of the geographical focus of the research conducted in the field of venture capital (RQ4). The studies on venture capital have focused primarily on a single country, whose share has gone from 42.41% between 2001 and 2006 to 48.83% between 2017 and 2021. The proportion of studies with multi-country samples has also grown from 22.57% between 2001 and 2006 to 32.97% between 2017 and 2021. In terms of studies focusing on only a single country, the USA has emerged as the most popular country for study among venture capital researchers. Nonetheless, the share of studies on China has also increased over time. Yet, most studies in the field remain predominantly focused on the west, with America and Europe garnering the most attention among researchers, though the shares of Asian and African countries have also risen over the years.

**Table 5:** Geographical focus of venture capital research

	2001–2006	2007–2011	2012–2016	2017–2021	Total
**Panel A: Country share**					
Single country	42.41%	42.17%	46.55%	48.83%	45.74%
Multi country	22.57%	30.12%	34.24%	32.97%	30.97%
No geographical data reported	35.02%	27.71%	19.21%	18.20%	23.29%
**Panel B: Country**					
USA	21.79%	23.19%	24.38%	23.78%	23.48%
China	0.78%	1.81%	4.93%	12.61%	6.32%
UK	3.11%	2.41%	2.96%	2.16%	2.58%
Germany	4.67%	3.61%	1.48%	1.08%	2.32%
Canada	2.33%	1.20%	1.23%	0.90%	1.29%
Spain	0.00%	0.60%	2.22%	0.72%	0.97%
Sweden	1.17%	1.20%	0.74%	0.72%	0.90%
India	1.95%	0.30%	0.49%	0.90%	0.84%
Japan	1.17%	0.00%	0.74%	0.72%	0.65%
Australia	1.17%	1.20%	0.00%	0.36%	0.58%
Israel	0.78%	0.90%	0.74%	0.18%	0.58%
Italy	0.00%	1.20%	0.00%	0.90%	0.58%
Belgium	0.39%	0.30%	0.99%	0.18%	0.45%
Poland	0.78%	0.60%	0.49%	0.00%	0.39%
South Korea	0.39%	0.90%	1.23%	0.72%	0.84%
Singapore	0.78%	0.30%	0.25%	0.18%	0.32%
France	0.39%	0.00%	0.49%	0.18%	0.26%
Netherlands	0.00%	0.00%	0.49%	0.36%	0.26%
Taiwan	0.39%	0.90%	0.00%	0.00%	0.26%
Chile	0.00%	0.30%	0.25%	0.18%	0.19%
Portugal	0.00%	0.00%	0.49%	0.18%	0.19%
South Africa	0.00%	0.30%	0.25%	0.18%	0.19%
Ireland	0.39%	0.00%	0.00%	0.18%	0.13%
Bangladesh	0.00%	0.00%	0.00%	0.18%	0.06%
Brazil	0.00%	0.00%	0.25%	0.00%	0.06%
Czech Republic	0.00%	0.00%	0.00%	0.18%	0.06%
England	0.00%	0.00%	0.25%	0.00%	0.06%
Hong Kong	0.00%	0.00%	0.25%	0.00%	0.06%
Indonesia	0.00%	0.00%	0.00%	0.18%	0.06%
Jordan	0.00%	0.00%	0.00%	0.18%	0.06%
Latvia	0.00%	0.00%	0.25%	0.00%	0.06%
Lithuania	0.00%	0.30%	0.00%	0.00%	0.06%
Malaysia	0.00%	0.00%	0.00%	0.18%	0.06%
Mexico	0.00%	0.00%	0.25%	0.00%	0.06%
Nicaragua	0.00%	0.30%	0.00%	0.00%	0.06%
Norway	0.00%	0.30%	0.00%	0.00%	0.06%
Serbia	0.00%	0.00%	0.25%	0.00%	0.06%
Switzerland	0.00%	0.00%	0.00%	0.18%	0.06%
Tanzania	0.00%	0.00%	0.00%	0.18%	0.06%
Uganda	0.00%	0.00%	0.00%	0.18%	0.06%
Ukraine	0.00%	0.00%	0.25%	0.00%	0.06%

Panel B reports the most focused upon countries in single country studies

Table 6 shows the trend of methodological choices of venture capital researchers (RQ5). Panel A indicates that most studies in the field are empirical in nature, with its share growing from 61.09% between 2001 and 2006 to 79.10% between 2017 and 2021. Panel B suggests that studies in the field are mostly quantitative, with the share of qualitative studies declining over time. The data used in such studies tend to be archival in nature, with other data types having a small share as per Panel C. Taken collectively, venture capital researchers appear to favor a slant towards empirical, quantitative, and archival research.

**Table 6:** Methodological choice of venture capital research

	2001–2006	2007–2011	2012–2016	2017–2021	Total
**Panel A: Research approach**					
Empirical	61.09%	70.48%	78.33%	79.10%	74.06%
Conceptual	21.40%	17.77%	12.32%	11.53%	14.71%
Modelling and analytical	5.06%	2.71%	1.72%	2.88%	2.90%
Review	3.11%	2.71%	1.72%	3.60%	2.84%
Mixed	4.67%	2.71%	4.19%	1.80%	3.10%
Not reported	4.67%	3.61%	1.72%	1.08%	2.39%
**Panel B: Research design**					
Quantitative	56.03%	63.25%	72.91%	72.61%	67.94%
Qualitative	33.07%	27.71%	22.17%	23.06%	25.48%
Mixed	6.23%	5.42%	3.20%	3.42%	4.26%
Not reported	4.67%	3.61%	1.72%	0.90%	2.32%
**Panel C: Research data**					
Archival	48.25%	58.43%	69.46%	68.11%	63.10%
Survey	7.78%	7.53%	3.69%	3.96%	5.29%
Case study	3.50%	4.52%	4.93%	3.24%	4.00%
Interview	2.72%	3.01%	1.23%	2.52%	2.32%
Experimental	0.78%	0.60%	0.25%	1.44%	0.84%
Mixed	1.56%	1.51%	1.48%	2.88%	2.00%
No data reported	35.41%	24.40%	18.97%	17.84%	22.45%

## Science mapping

### Co-authorship analysis

**Fig. 5:**
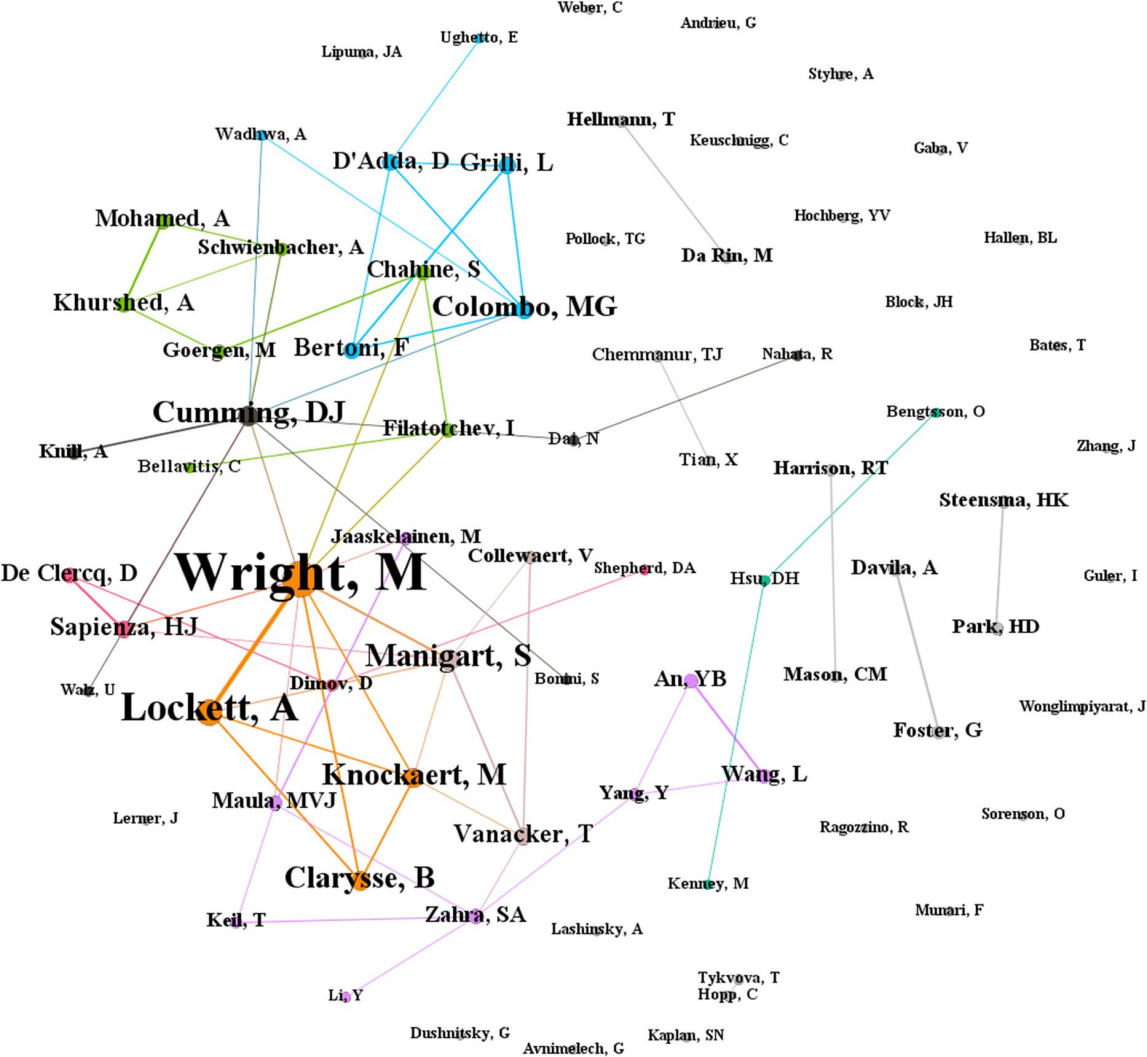
Co-authorship network of venture capital researchers

As part of our endeavor to achieve our third research objective, we conducted a co-authorship analysis (RQ6). Using this method, we identify several major author groups who have contributed and shaped the field of venture capital. The network of co-authorship is constructed for authors who have contributed at least five publications in the field. The analysis resulted in numerous clusters, wherein only eight clusters had three or more authors—we focus on these major clusters in this study. Figure 5 presents the collaboration network of authors while the summary of author groups is presented in Table 7.

**Table 7:** Summary of prominent author groups for venture capital research

Author group	Author	Total link strength	Average publication year	Thematic focus	Geographical focus
#1	Markku V. J. Maula	6	2009.57	• Venture capital networks • Venture capital portfolios	• USA • China
	Yunbi An	6	2018.83		
	Lei Wang	6	2018.50		
	Shaker A. Zahra	6	2010.00		
	Mikko Jaaskelainen	4	2011.20		
	Thomas Keil	3	2008.00		
	Yi Yang	3	2010.67		
	Yong Li	1	2011.89		
#2	Arif Khurshed	8	2014	• Venture capital syndication • Initial public offerings	• Europe
	Salim Chahine	7	2014.83		
	Abdulkadir Mohamed	7	2016.33		
	Igor Filatotchev	6	2015.71		
	Armin Schwienbacher	5	2012.33		
	Marc Goergen	5	2011.40		
	Cristiano Bellavitis	2	2019.25		
#3	Massimo G. Colombo	11	2015.80	• Venture capital growth and performance	• Europe • Italy
	Luca Grilli	9	2015.12		
	Fabio Bertoni	9	2014.43		
	Diego D'Adda	8	2017.67		
	Anu Wadhwa	2	2013.00		
	Elisa Ughetto	1	2015.00		
#4	Douglas Cumming	13	2010.20	• Entrepreneurial finance	• Europe
	April Knill	4	2014.00		
	Na Dai	2	2011.80		
	Rajarishi Nahata	1	2012.83		
	Uwe Walz	1	2009.17		
	Stefano Bonini	1	2016.00		
#5	Mike Wright	32	2008.32	• Venture capital syndication • University spinouts • Decision making in venture capital firms	• Europe • UK
	Andy Lockett	20	2005.50		
	Bart Clarysse	12	2008.67		
	Mirjam Knockaert	12	2009.20		
#6	Harry J. Sapienza	9	2007.29	• Venture capital investments	• USA • Europe
	Dirk De Clercq	7	2007.71		
	Dimo Dimov	3	2008.71		
	Dean A. Shepherd	1	2006.50		
#7	David H. Hsu	2	2008.50	• Entrepreneurial finance	• USA
	Ola Bengtsson	1	2012.86		
	Martin Kenney	1	2009.86		
#8	Sophie Manigart	14	2010.73	• Venture capital investment decisions	• Europe
	Tom Vanacker	9	2014.67		
	Veroniek Collewaert	4	2016.17		

### Author group #1: Maula et al.

The largest author group consisting of eight authors is led by Markku V. J. Maula, who has the highest total link strength. Geographically, the concentration of these authors has been predominantly in China and the USA, with the thematic focus being on venture capital networks and portfolios. The authors have also worked on institutional research across multiple countries. The average publication year of this author group is 2012, with the publications by Yunbi An being the most recent at an average publication year of 2018.

### Author group #2: Khurshed et al.

The second largest author group containing seven authors is led by Arif Khurshed, who has the highest total link strength. Geographically, these authors focus on multi-country studies, with a predominant focus on Europe. Thematically, venture capital syndication and initial public offerings are some of the noteworthy contributions by this author group.

### Author group #3: Colombo et al.

The joint third-largest author group, which consists of six authors, is led by Massimo Colombo. The average publication year of this author group is 2015, indicating that the publications from this author group appear around the same time as the second largest author group, whose average publication year is also 2015. Both the second and third largest author groups are much ‘younger’ than the largest author group. Thematically, the authors in this group have focused on venture capital growth and performance, whereas geographically, the group seems to have a European focus, with particular interest on Italy.

### Author group #4: Cumming et al.

The next joint third-largest author group, which also consists of six authors, is led by Douglas Cumming. The thematic focus of this author group has been on entrepreneurial finance while their geographic interest has been multi-country in Europe. The average publication year of this author group is 2012. This represents a potential timeline for the emergence of entrepreneurial finance research in Europe.

### Author group #5: Wright et al.

This author group, though consisting of only four authors, is arguably the most important author group for venture capital research. This assertion is predicated on the prominence of this author group in the collaboration network (Fig. 2) and the prolific publication of all four authors in this author group, all of whom appear in the list of the top most prolific authors (Table 2). Furthermore, the connection between these authors is notably strong, indicating their repeated collaborations with one another. Their thematic range is also wide, with a generous focus on topics such as venture capital syndication, university spinouts, and decision-making in venture capital. The geographical focus of this author group is on Europe, with the UK receiving much attention. However, the average publication years of these authors range between 2005 and 2009, which indicates that these authors are less prolific in recent times. However, the works of these four authors remain significant in the field.

One of the main authors in this group, Mike Wright, passed away in 2019. This is a big loss to the academic community, as Mike Wright was most cited in both venture capital and private equity on Google Scholar (see Appendix). Mike Wright also established the Centre for Management Buyout Research at the University of Nottingham in 1986, which offers a leading source of data and information on buyouts as well as venture capital, with a focus on Europe. The *British Journal of Management* (*BJM*) advertised a call for papers to honor Mike Wright’s contributions to entrepreneurial finance shortly after his passing in 2019. *BJM* published this special issue on entrepreneurial finance in Mike Wright’s honor in 2022 (Budhwar et al., 2022).

### Author group #6: Sapienza et al.

This author group is another one that is “older,” with its authors’ average publication years falling between 2006 and 2009. The group is led by Harry J. Sapienza and the thematic focus of the group has been on the determinants of venture capital investments. Geographically, this author group has a multi-country focus on Europe and the USA.

### Author group #7: Hsu et al.

The author group, which is smaller than the other author groups, is led by David H. Hsu. Thematically, the focus of this author group is on entrepreneurial finance, whereas geographically, their focus is predominantly on the USA. The average publication years of the authors in this group range between 2008 and 2013, making this cluster ‘older’ than the fourth author group. This could indicate that entrepreneurial finance research in the USA emerged earlier than that in Europe. However, more research is required to bolster this assertion.

### Author group #8: Manigart et al.

This author group is another one of the ‘younger’ author groups, with average publication years ranging between 2010 and 2016. The group is led by Sophie Manigart. Thematically, this author group concentrates on venture capital investment decisions. Geographically, the research of this author group tends to be multi-country in Europe.

#### Co-citation analysis

To find the foundational themes in venture capital research (RQ7), we use co-citation analysis. The co-citation analysis has been established as a valid means of study in a scientific discipline and is instrumental in identifying the intellectual structure of a field (Ramos-Rodrígue & Ruíz-Navarro, 2004). The premise here is that authors often draw from each other’s works in addition to drawing from common sources of knowledge (Nerur et al., 2008). The citation is often a form of intellectual dependence (Culnan, 1987), where one work draws build upon the knowledge created in the works that came before. The co-citation of two papers occurs when they are cited together in a third document, indicating intellectual similarity (Small, 1973). The co-citation thus focuses on the works that are cited in the paper, rather than the paper itself. Here, the co-citation analysis of the most cited references by venture capital research revealed 422 articles that can be segmented into five clusters, with each cluster representing a foundational theme in the field of venture capital. The analysis is based on local citations (i.e., the number of times a reference appears in the reference list of the articles in the corpus), which indicate the impact of any reference on venture capital research in the corpus. Table 8 presents the summary of the foundational themes in venture capital research.

**Table 8:** Prominent themes in venture capital research

Theme	Author(s)	Title	Source	Year	TC
**Theme #1: Venture capital adoption and financing processes** (TP: 122; TC: 4,999)					
**Most cited article**	Gompers, P; Lerner, J	The venture capital cycle	MIT Press, Cambridge	1999	288
	Sorenson, O; Stuart, TE	Syndication networks and the spatial distribution of venture capital investments	*American Journal of Sociology*	2001	231
	Stuart, TE; Hoang, H; Hybels, RC	Interorganizational endorsements and the performance of entrepreneurial ventures	*Administrative Science Quarterly*	1999	164
	Cohen, WM; Levinthal, DA	Absorptive capacity: A new perspective on learning and innovation	*Administrative Science Quarterly*	1990	93
	Dushnitsky, G; Lenox, MJ	When do incumbents learn from entrepreneurial ventures?: Corporate venture capital and investing firm innovation rates	*Research Policy*	2005	92
**Key topics**	• Venture capital investments • Venture capital process • Funding cycles				
**Theme #2: Venture capital roles in business** (TP: 118; TC: 4,614)					
**Most cited article**	Gorman, M; Sahlman, WA	What do venture capitalists do?	*Journal of Business Venturing*	1989	189
	Hsu, DH	What do entrepreneurs pay for venture capital affiliation?	*Journal of Finance*	2004	183
	Baum, JAC; Silverman, BS	Picking winners or building them? Alliance, intellectual, and human capital as selection criteria in venture financing and performance of biotechnology startups	*Journal of Business Venturing*	2004	131
	Sapienza, HJ; Manigart, S; Vermeir, W	Venture capitalist governance and value added in four countries	*Journal of Business Venturing*	1996	116
	Sapienza, HJ	When do venture capitalists add value?	*Journal of Business Venturing*	1992	113
**Key topics**	• Value addition through venture capital • Effect of venture capital funds on governance and strategy of firms				
**Theme #3: Venture capital governance** (TP: 110; TC: 6,165)					
**Most cited article**	Sahlman, WA	The structure and governance of venture-capital organizations	*Journal of Financial Economics*	1990	297
	Hellmann, T; Puri, M	Venture capital and the professionalization of start-up firms: Empirical evidence	*Journal of Finance*	2002	269
	Gompers, PA	Optimal investment, monitoring, and the staging of venture capital	*Journal of Finance*	1995	242
	Kaplan, SN; Strömberg, P	Financial contracting theory meets the real world: An empirical analysis of venture capital contracts	*Review of Economic Studies*	2003	196
	Lerner, J	Venture capitalists and the oversight of private firms	*Journal of Finance*	1995	192
**Key topics**	• Governance and strategy in venture capital organizations • Effect of venture capital on firm competencies				
**Theme #4: Venture capital syndication** (TP: 39; TC: 1,855)					
**Most cited article**	Hochberg, YV; Ljungqvist, A; Lu, Y	Whom you know matters: Venture capital networks and investment performance	*Journal of Finance*	2007	244
	Lerner, J	The syndication of venture capital investments	*Financial Management*	1994	191
	Brander, JA; Amit, R; Antweiler, W	Venture-capital syndication: Improved venture selection vs. the value-added hypothesis	*Journal of Economics and Management Strategy*	2002	139
	Wright, M; Lockett, A	The structure and management of alliances: Syndication in the venture capital industry	*Journal of Management Studies*	2003	86
	Bygrave, WD	Syndicated investments by venture capital firms: A networking perspective	*Journal of Business Venturing*	1987	72
**Key topics**	• Venture capital syndication • Cross border venture capital networks				
**Theme #5: Venture capital and creation of public organizations** (TP: 33; TC: 1,805)					
**Most cited article**	Megginson, WL; Weiss, KA	Venture capitalist certification in initial public offerings	*Journal of Finance*	1991	198
	Gompers, PA	Grandstanding in the venture capital industry	*Journal of Financial Economics*	1996	150
	Barry, CB; Muscarella, CJ; Peavy III, JW; Vetsuypens, MR	The role of venture capital in the creation of public companies. Evidence from the going-public process	*Journal of Financial Economics*	1990	129
	Nahata, R	Venture capital reputation and investment performance	*Journal of Financial Economics*	2008	102
**Key topics**	• Venture capital certification • Structure and governance of venture capital alliances				

### Theme #1: Venture capital adoption and financing processes

This is the largest cluster formed with 122 cited references. The central theme of this cluster is the adoption and financing process of venture capital, with topics such as the venture capital cycle, the effect of venture capital on the performance of entrepreneurial ventures, the strategy in business backed by venture capital, as well as the institutional factors affecting them. This cluster also highlights the impact of venture capital on firms. Authors such as Gompers and Lerner (1999) present a comprehensive overview of the venture capital cycle, whereas Sorenson and Stuart (2001) reveal the effect of the interfirm network on shaping venture capital investments, and Stuart et al., (1999) show the effect of interorganizational networks on firm performance. Noteworthily, the contributions of these authors inherently concentrate on the adoption of venture capital, with the authors exploring “how” such investments take place and impact firms, thereby contributing to the field’s foundational understanding. Other firm-level characteristics such as absorptive capacity (Cohen & Levinthal, 1990), competitive advantage (Barney, 1991), corporate alliance (Dushnitsky & Lavie, 2010), innovation (Dushnitsky & Lenox, 2005a, 2005b), and knowledge management (Dushnitsky & Shaver, 2009; Wadhwa & Kotha, 2006), as well as institutional influences (Guler, 2007), also receive attention in this cluster.

### Theme #2: Venture capital roles in business

The central theme of the second largest cluster concentrates on venture capital itself, specifically on the roles of venture capital in business. As one of the primary sources of financing and leverage for startups, early-stage, and emerging companies that have high growth potential, the value that venture capitalists contribute, both monetarily and non-monetary, is important. While public organizations receive scrutiny from regulators and the public, the same cannot be said about other firms, especially newer firms, and thus, venture capital investors can serve the same purpose through the governance of newer firms, which highlights the added value that venture capital investors can bring through advisory and monitoring. This is in addition to the experience and leverage that venture capital can offer to firms that receive their investment. These key roles are exemplified through the studies in this cluster, which have explored the roles of venture capital in business (Gorman & Sahlman, 1989; Sapienza et al., 1996), and the value added to firms as a result of the involvement of venture capital investors in firms (Baum & Silverman, 2004; Hsu, 2004; Sapienza, 1992). The understanding of the role that venture capital can play in business has also been studied in tandem with the determinants of venture capital investments (Gupta & Sapienza, 1992), the decision-making process in venture capital investments (Macmillan et al., 1985, 1989), the modelling of venturing capital decision making (Tyebjee & Bruno, 1984), and role of government venture capital on young and innovative firms (Colombo et al., 2016).

### Theme #3: Venture capital governance

The third largest cluster deals with a foundational theme on venture capital governance. The studies in this cluster demonstrate that governance is important to both venture capital firms and the firms that receive venture capital investment. Though the contribution of the present cluster appears to overlap with the previous cluster, a noteworthy observation is that this cluster devotes itself to the manifestation of governance in venture capital, whereas the previous cluster is broader and includes the multiple roles that venture capital can play in business, wherein governance plays a peripheral (i.e., topic) rather than a central (i.e., theme) role. Sahlman (1990) describes the structure and governance of venture capital firms, whereas Gompers (1995) explores the optimal investment, monitoring, and staging of venture capital. Other scholars such as Hellmann and Puri (2002) concentrate on venture capital firms in relation to the development and professionalization of new firms, whereas Kaplan and Stromberg (2003) and Cumming and Johan (2013) focus on venture capital contracts, and Lerner (1995) on the effect of venture capital investors on firm oversight. Other studied topics include the role of venture capital in firm innovation (Kortum & Lerner, 2000), structure of capital markets (Black & Gilson, 1998), and private equity performance (S. N. Kaplan & Schoar, 2005).

### Theme #4: Venture capital syndication

This second smallest cluster deals with the theme on venture capital syndication, including its alliances and networks. The studies in this cluster have focused on explaining the structure, innerworkings, and impact of venture capital networks. Hochberg et al., (2007) argue that whom you know matters in their exploration of the relationship between venture capital networks and investment performance, whereas Lerner (1994) and Brander et al., (2002) shed light on the syndication of venture capital investments and its impact on added value and venture selection. Other studies reveal the factors influencing the formation of venture capital alliances (Bygrave, 1987) and the structure and management of such alliances (Wright & Lockett, 2003). Their findings suggest that venture capital investments are primarily driven by the potential and volatility of returns, which motivate venture capital firms to diversify risk by way of syndicated and joint investments, highlighting the importance of the management of such networks in the process. Also discussed in this cluster is the specialization and diversification of venture capital funds (Norton & Tenenbaum, 1993). Noteworthily, the study of structures of venture capital, including its syndication, is highly important to gain a comprehensive understanding of the venture capital industry and its inherent decision-making processes. Therefore, this foundational theme, despite being relatively small in its publications, remains central to the understanding of venture capital investments.

### Theme #5: Venture capital and creation of public organizations

The fifth and final foundational theme deals with the role of venture capital investors in the creation of public organizations. The studies in this cluster concentrate on the certification role of venture capital investors in IPOs (Barry et al., 1990; Megginson & Weiss, 1991) and the development of venture capital firms (P. A. Gompers, 1996) and their reputation (Nahata, 2008). The effect of venture capital investors on firm funding and IPOs has been explored at length in this cluster. Nonetheless, this is the smallest cluster, which indicates that this foundational theme has received lesser attention as compared to the other foundational themes. The publication year of the references constituting this cluster averages at 1995, which indicates that this foundational theme serves as the basis for much of the discussion in venture capital research. In comparison, the average publication years of the references constituting the first, second, third, and fourth clusters are 1998, 2000, 2003, and 2004, respectively, indicating that the fifth cluster is the oldest cluster among the foundational clusters of venture capital research.

#### Emergent research frontiers in venture capital research

In order to locate the emerging themes in venture capital research (RQ7), we use bibliographic coupling. The application of bibliographic coupling on the articles published in the last three years at the time this review was conducted (2019–2021) led to the creation of several clusters, wherein seven were major clusters as they covered approximately 97% of the total publications on venture capital during the studied period (361 out of 374). These clusters represent the major frontiers of the field as they have been explored most prominently and recently by researchers. The clusters are also ordered from the largest to the smallest in terms of total publications and reviewed to define their central themes. Noteworthily, there is connectivity across themes, which is reasonably expected as they belong to the same field of research. In this regard, a theme could be tangentially discussed in tandem with another theme. Therefore, the connected nature of research in the field should be taken into account when interpreting the nuances and trajectory of venture capital research. Table 9 presents a summary of the emergent frontiers in the field.

**Table 9:** Emergent research frontiers in venture capital research

Frontier	Author(s)	Title	Year	Source	TC
**Frontier #1: Venture capital and sustainable entrepreneurship** (TP: 94, TC: 462)					
**Most cited article**	Chowdhury, F; Audretsch, DB; Belitski, M	Institutions and entrepreneurship quality	2019	*Entrepreneurship Theory and Practice*	56
	Guzman, J; Kacperczyk, A	Gender gap in entrepreneurship	2019	*Research Policy*	47
	Howell, ST; Niessner, M; Yermack, D	Initial coin offerings: Financing growth with cryptocurrency token sales	2020	*Review of Financial Studies*	37
	Demirel, P; Danisman, GO	Eco-innovation and firm growth in the circular economy: Evidence from European small- and medium-sized enterprises	2019	*Business Strategy and the Environment*	35
	Pan, FH; Yang, BF	Financial development and the geographies of startup cities: Evidence from China	2019	*Small Business Economics*	26
**Future research**	• How can venture capital investors select or nurture economically, environmentally, and socially conscious enterprises? • How can venture capital investors and their investments contribute to the sustainable development goals?				
**Frontier #2: Fintech and crowdfunding** (TP: 60, TC: 525)					
**Most cited article**	Haddad, C; Hornuf, L	The emergence of the global fintech market: Economic and technological determinants	2019	*Small Business Economics*	121
	Vismara, S	Sustainability in equity crowdfunding	2019	*Technological Forecasting and Social Change*	88
	Cumming, D; Meoli, M; Vismara, S	Investors’ choices between cash and voting rights: Evidence from dual-class equity crowdfunding	2019	*Research Policy*	68
	Brown, R; Rocha, A; Cowling, M	Financing entrepreneurship in times of crisis: Exploring the impact of COVID-19 on the market for entrepreneurial finance in the United Kingdom	2020	*International Small Business Journal-Researching Entrepreneurship*	65
	Cumming, D; Meoli, M; Vismara, S	Does equity crowdfunding democratize entrepreneurial finance?	2021	*Small Business Economics*	59
	Ahluwalia, S; Mahto, RV; Guerrero, M	Blockchain technology and startup financing: A transaction cost economics perspective	2020	*Technological Forecasting and Social Change*	56
**Future research**	• How does changes in the technological environment affect the changes in business models and the source of financing options available to firms? • How can firms access to democratized ways of technology-enabled financing, and what can they look forward to (e.g., opportunities) and should look out for (i.e., pitfalls) in a democratized financial market empowered by technology? • What are the antecedents and consequences of contemporary and democratized financing for both investors and investments, and what are its similarities and differences as compared to traditional financing?				
**Frontier #3: Venture capital investment strategies** (TP: 53, TC: 187)					
**Most cited article**	Gomulya, D; Jin, K; Lee, PM; Pollock, TG	Crossed wires: Endorsement signals and the effects of IPO firm delistings on venture capitalists’ reputations	2019	*Academy of Management Journal*	15
	Nazareno, J; Zhou, M; You, TL	Global dynamics of immigrant entrepreneurship Changing trends, ethnonational variations, and reconceptualizations	2019	*International Journal of Entrepreneurial Behavior and Research*	14
	Block, JH; Fisch, CO; Obschonka, M; Sandner, PG	A personality perspective on business angel syndication	2019	*Journal of Banking and Finance*	13
	Conti, A; Dass, N; Di Lorenzo, F; Graham, SJH	Venture capital investment strategies under financing constraints: Evidence from the 2008 financial crisis	2019	*Research Policy*	12
	Amornsiripanitch, N; Gompers, PA; Xuan, YH	More than money: Venture capitalists on boards	2019	*Journal of Law, Economics and Organization*	8
**Future research**	• What non-financial aspects in a firm do venture capital investors find attractive, may consider, or will look for when making funding and investment decisions? • How do venture capital investors evaluate a firm’s ability to achieve non-financial objectives, and how are they similar or different to that for financial objectives? • How can firms seeking venture capital funding and investment leverage on new-age practices (e.g., ESG) and technologies (e.g., big data analytics) and innovate to deliver on both financial and non-financial aspects of performance expected by investors?				
**Frontier #4: Venture capital and innovation** (TP: 50, TC: 157)					
**Most cited article**	Guo, B; Perez-Castrillo, D; Toldra-Simats, A	Firms’ innovation strategy under the shadow of analyst coverage	2019	*Journal of Financial Economics*	20
	Kim, JY; Steensma, HK; Park, HD	The influence of technological links, social ties, and incumbent firm opportunistic propensity on the formation of corporate venture capital deals	2019	*Journal of Management*	11
	Pan, LL; Li, XM; Chen, JH; Chen, TX	Sounds novel or familiar? Entrepreneurs’ framing strategy in the venture capital market	2020	*Journal of Business Venturing*	10
	Rohm, P; Merz, M; Kuckertz, A	Identifying corporate venture capital investors—A data-cleaning procedure	2020	*Finance Research Letters*	9
	Rossi, M; Festa, G; Devalle, A; Mueller, J	When corporations get disruptive, the disruptive get corporate: Financing disruptive technologies through corporate venture capital	2020	*Journal of Business Research*	9
**Future research**	• What do venture capital investors consider ‘innovative’, and what cues of innovation do they look for in firms when making funding and investment decisions? • How do venture capital investment returns differ across the various forms of innovation (e.g., incremental, new to the word), and to what extent do factors such as technology influence investment performance and returns?				
**Frontier #5: Entrepreneurial finance** (TP: 41, TC: 130)					
**Most cited article**	Colombo, MG.; D’Adda, D; Quas, A	The geography of venture capital and entrepreneurial ventures’ demand for external equity	2019	*Research Policy*	15
	Gornall, W; Strebulaev, IA	Squaring venture capital valuations with reality	2020	*Journal of Financial Economics*	14
	Babich, V; Marinesi, S; Tsoukalas, G	Does crowdfunding benefit entrepreneurs and venture capital investors?	2021	*Manufacturing and Service Operations Management*	13
	Lerner, J; Nanda, R	Venture capital’s role in financing innovation: What we know and how much we still need to learn	2020	*Journal of Economic Perspectives*	10
	Wu, L; Xu, L	Venture capital certification of small and medium-sized enterprises towards banks: Evidence from China	2020	*Accounting and Finance*	11
**Future research**	• What is the psychological process that underpins the decision making of venture capital investors, and how does this process differ and interact with the rational process? • How does irrational/impulsive decision making of venture capital investors affect the returns of the entrepreneurial ventures that they invest in, and to what extent do they differ from rational/planned decision making?				
**Frontier #6: Venture capital and IPOs** (TP: 40, TC: 77)					
**Most cited article**	Megginson, WL; Meles, A; Sampagnaro, G; Verdoliva, V	Financial distress risk in initial public offerings: How much do venture capitalists matter?	2019	*Journal of Corporate Finance*	13
	Sakawa, H; Watanabel, N	IPO underpricing and ownership monitoring in Japan	2020	*Asian Business and Management*	6
	Chahine, S; Saade, S; Goergen, M	Foreign business activities, foreignness of the VC syndicate, and IPO value	2019	*Entrepreneurship Theory and Practice*	6
	Li, EM; Liao, L; Wang, ZW; Xiang, HY	Venture capital certification and customer response: Evidence from P2P lending platforms	2020	*Journal of Corporate Finance*	5
	Ozmel, U; Trombley, TE; Yavuz, MD	Outside insiders: Does access to information prior to an IPO generate a trading advantage after the IPO?	2019	*Journal of Financial and Quantitative Analysis*	4
**Future research**	• What mechanisms can venture capital leverage to influence IPO performance? • To what extent can venture capital influence IPO performance across different economic conditions (e.g., financial distress)?				
**Frontier #7: Drivers of venture capital funding decisions** (TP: 23, TC: 110)					
**Most cited article**	Tian, XL; Kou, G; Zhang, WK	Geographic distance, venture capital and technological performance: Evidence from Chinese enterprises	2020	*Technological Forecasting and Social Change*	22
	Gou, XJ; Liao, HC; Wang, XX; Xu, ZS; Herrera, F	Consensus based on multiplicative consistent double hierarchy linguistic preferences: Venture capital in real estate market	2020	*International Journal of Strategic Property Management*	18
	Liu, XD; Wang, ZW; Zhang, ST; Liu, JS	Probabilistic hesitant fuzzy multiple attribute decision-making based on regret theory for the evaluation of venture capital projects	2020	*Economic Research-Ekonomska Istrazivanja*	14
	Zhang, WK; Tian, XL; Yu, A	Is high-speed rail a catalyst for the fourth industrial revolution in China? Story of enhanced technology spillovers from venture capital	2020	*Technological Forecasting and Social Change*	10
	de Leeuw, T; Gilsing, V; Duysters, G	Greater adaptivity or greater control? Adaptation of IOR portfolios in response to technological change	2019	*Research Policy*	9
**Future research**	• How do the behavioral and psychological profile of venture capital investors affect their funding decisions? • How does disruptive changes, externalities, and social sentiments affect funding decisions among venture capital investors? • How do funding decisions differ among venture capital investors of different generations?				

### Frontier #1: Venture capital and sustainable entrepreneurship

The largest frontier concentrates on sustainable entrepreneurship. The authors contributing to research in this frontier have explored venture capital in tandem with the role of institutions in fostering entrepreneurship quality (Chowdhury et al., 2019), gender gaps in entrepreneurship (Guzman & Kacperczyk, 2019), IPOs (Howell et al., 2020), eco-innovation and firm growth in the circular economy (Demirel & Danisman, 2019), the financial development of startup cities (F. Pan & Yang, 2019), and the role of environmental policies in spurring venture capital (Bianchini & Croce, 2022). This frontier appears to be motivated in part by the United Nations Sustainable Development Goals, which have led to governments around the world striving to follow the path of sustainable development, thereby formulating and implementing policies targeted at achieving economically, environmentally, and socially responsible development. This seems to have affected venture capital investment and enterprise selection, and thus this theme’s development. Noteworthily, there is a strong sense of economically, environmentally, and socially conscious entrepreneurship spearheaded by venture capital-backed sustainable enterprises. This emerging interest lays a path forward for the future, with calls for new research relating to the following research questions:

- *How can venture capitalists select or nurture economically, environmentally, and socially conscious enterprises?*
- *How can venture capitalists and their investments contribute to the sustainable development goals?*

### Frontier #2: Fintech and crowdfunding

The second largest frontier deals with fintech and crowdfunding, with authors exploring topics related to the emergence of fintech (Haddad & Hornuf, 2019), crowdfunding (Brown et al., 2019a, 2019b, 2020; Cumming et al., 2019a, 2019b, 2019c, 2021a, 2021b; Johan & Zhang, 2021b; Vismara, 2019), and blockchain (or the technology empowering fintech and crowdfunding) (Ahluwalia et al., 2020). Apart from this, other studies have also shed light on digital entrepreneurship (Cavallo et al., 2019) and technology parks (Cumming & Zhang, 2019; Robinson, 2022), including their role in the development of new-age financing. There has also been a focus on sustainability (Vismara, 2019), and separation of ownership and control (Cumming et al., 2019a, 2019b, 2019c) in relation to their effects on the success or failure of crowdfunding campaigns. Noteworthily, authors are investing their focus on the more ‘democratized’ ways of financing such as crowdfunding and initial coin offerings (Cumming et al., 2021a, 2021b), which have become increasingly popular with time (Butticè & Vismara, 2022). Specifically, the upheavals in the financial world due to successive crisis (e.g., economic, public health—e.g., COVID-19) and the emergence of new business models driven by the fourth industrial revolution (e.g., blockchain, internet of things) have led to a shift in all aspect of conducting business including the way they are financed. With information and innovation being democratized due to the internet and with digital communities having a great influence over the flow the knowledge today than in the past, it is expected that the financing of business too will change and therefore the topics of fintech and internet-based funding such as crowdfunding seem to have emerged to transplant, in some part at least, the traditional sources of financing. This seems to be the reason why research in this area has emerged and proliferated in recent times, and thus, holds the potential for leading a way to the future for more research on the democratized ways of financing and the changes in financing models that have been influenced by contemporary changes over time.

- *How does changes in the technological environment affect the changes in business models and the source of financing options available to firms?*
- *How can firms access to democratized ways of technology-enabled financing, and what can they look forward to (e.g., opportunities) and should look out for (e.g., pitfalls) in a democratized financial market empowered by technology?*
- *What are the antecedents and consequences of contemporary and democratized financing for both investors and investments, and what are its similarities and differences as compared to traditional financing?*

### Frontier #3: Venture capital investment strategies

The third largest frontier focuses on investment strategies in the field of venture capital. The authors contributing to this frontier concentrate on venture capital endorsements (Gomulya et al., 2019), new trends in entrepreneurship (e.g., immigrant entrepreneurship) (Nazareno et al., 2019), syndication of angel investments (J. H. Block et al., 2019a, 2019b), the effect of financial constraints on investment strategies (Conti et al., 2019), and the factors influencing venture capital roles in the board of companies (Amornsiripanitch et al., 2019). They also explore the investment strategies adopted by venture capital including syndication (Luo et al., 2019), and partner selection (Cheng & Tang, 2019), as well as the effect of venture capital on firm outcomes such as innovation (Que & Zhang, 2020) and reputation (Chahine et al., 2021a, 2021b). The effect of venture capital investors on firms is one of the foundational themes in the field, and its continuation in recent times reflects the importance of this theme. The field has nonetheless gone beyond financial performance as it now includes the non-financial performance of firms. This indicates a shift in the thinking of researchers who no longer look at firm performance the same way they used to in earlier times. This raises several potentially interesting and fruitful research questions for future research:

- *What non-financial aspects in a firm do venture capital investors find attractive, may consider, or will look for when making funding and investment decisions?*
- *How do venture capital investors evaluate a firm’s ability to achieve non-financial objectives, and how are they similar or different to that for financial objectives?*
- *How can firms seeking venture capital funding and investment leverage on new-age practices (e.g., ESG) and technologies (e.g., big data analytics) and innovate to deliver on both financial and non-financial aspects of performance expected by investors?*

### Frontier #4: Venture capital and innovation

The fourth largest research frontier is dedicated to entrepreneurial ventures and innovation. The topics explored as part of this frontier include the innovation strategy of firms (Guo et al., 2019), the technologies that drive collaboration among firms (Kim et al., 2019), the entrepreneurial and linguistic strategies that firms rely upon to deal with venture capital investors (L. Pan et al., 2019), and the roles of venture capital in the development of disruptive technologies (Rossi et al., 2020). The innovations by new firms are usually framed as new opportunities for venture capital investors. By investing in innovative firms, venture capital investors get more opportunities for higher returns, while firms secure the funding they need to develop and market their innovations. While the theme of the present frontier is related to the theme of the previous frontier, it should be noted that innovation takes center stage here as compared to its peripheral role in the other frontier. Nonetheless, new research in this space remains necessary in tandem with today’s marketplace characterized by high competition and rapid technological advancement. Thus, future research is encouraged, as follows:

- *What do venture capital investors consider ‘innovative’, and what cues of innovation do they look for in firms when making funding and investment decisions?*
- *How do venture capital investment returns differ across the various forms of innovation (e.g., incremental, new to the word), and to what extent do factors such as technology influence investment performance and returns?*

### Frontier #5: Entrepreneurial finance

The fifth largest research frontier concentrates on entrepreneurial finance. The topics explored as part of this frontier include the role of geographical distance between venture capital and entrepreneurs seeking external financing (Colombo et al., 2019), the valuation of venture capital investments in entrepreneurial ventures (Gornall & Strebulaev, 2020), the outcomes of investments for entrepreneurs and venture capital under the crowdfunding model (Babich et al., 2020), the role of venture capital in financing entrepreneurial innovations (Lerner & Nanda, 2020), and venture capital certification (Wu & Xu, 2020). Noteworthily, this research frontier highlights the importance of acknowledging and understanding new methods of entrepreneurial finance (Block et al., 2018). Specifically, research in this frontier not only focuses on funding by venture capital investors but also on their role in the firm acquisition of bank lending. However, much of the current research in this frontier is economic-focused, with little insight into the psychological process behind funding decisions for entrepreneurial ventures. In other words, current research is largely based on the assumption that venture capital investors are rational beings with all their investments thoughtfully planned, which ignores the irrational and speculative behavior exhibited by investors in the real world. Moving forward, we encourage researchers to explore the role of psychological processes and impulsive investment decisions, and to analyze how such investments turn out. Thus, the following research questions are proposed:

- *What is the psychological process that underpins the decision-making of venture capital investors, and how does this process differ and interact with the rational process?*
- *How does irrational/impulsive decision-making of venture capital investors affect the returns of the entrepreneurial ventures that they invest in, and to what extent do they differ from rational/planned decision-making?*

### Frontier #6: Venture capital and IPOs

The sixth largest research frontier deals with venture capital and IPOs, specifically the risk associated with IPOs and the impact of venture capital on IPO outcomes. The authors of this frontier have focused on topics such as financial distress (Megginson et al., 2019), IPO underpricing (Sakawa & Watanabel, 2020), factors affecting IPO value (Chahine et al., 2019), and trading advantages of IPOs (Ozmel et al., 2019). Noteworthily, this frontier highlights that venture capital backing and certification play a significant role in IPO performance. Future research on venture capital can take inspiration from this frontier and extend insights into this space through new explorations on the mechanisms through which venture capital affect IPO outcomes.

- *What mechanisms can venture capital leverage to influence IPO performance?*
- *What venture capital contractual terms influence the performance of a venture and enable a firm to go public?*
- *To what extent can venture capital influence IPO performance across different economic conditions (e.g., financial distress)?*
- *Why are venture capitalists better able to bring investee companies to IPOs in some countries than others?*

### Frontier #7: Drivers of venture capital funding decisions

The final research frontier is the smallest and deals with venture capital funding decisions and the drivers of such decisions. The exploration of topics in this frontier has been on the influence of geographical distance and technological performance (Tian et al., 2020), linguistic preferences (Gou et al., 2019), regret (Liu et al., 2020), technology spillovers (Zhang et al., 2020), and technological change (de Leeuw et al., 2019) on venture capital funding decisions. While decision-making models have been discussed in previous research frontiers and themes, the studies here are dedicated to decision-making from the venture capital investor perspective. Another difference is the discussion of decision-making models herein from the behavioral perspective with linguistic preferences and group decision-making gaining prominence. Nonetheless, the small size of this frontier indicates that research in this area is less developed. Thus, the following research questions are proposed to stimulate new research in this space:

- *How do the behavioral and psychological profiles of venture capital investors affect their funding decisions?*
- *How do disruptive changes, externalities, and social sentiments affect funding decisions among venture capital investors?*
- *How do funding decisions differ among venture capital investors of different generations?*
- *Why is there performance persistence across different venture capital fund managers?*

## Thematic analysis of private equity research

### Geographical focus and methodological choice analysis

To answer our fourth research question (RQ4), Table 10 presents an analysis of the geographical focus of private equity research. A plurality of the research in the field has been focused on single countries, which forms around 40% of all research. The multi-country studies are not far behind, though, comprising 33% of private equity studies. There has been an increase in the number of both single-country and multi-country studies throughout the period. Notably, studies that do not have a geographical focus (i.e., conceptual and review studies) have become less pronounced over time. Most single-country studies focus on the USA, followed by the UK. The share of studies focusing on the UK has decreased over time, however, and an increasing number of studies now focus on China. This indicates that in current research, scholars consider the institutional contexts of the USA and China to be more important. This may be because the USA and China are the two largest economies in the world. Furthermore, the USA and China also represent opposite sides of the spectrum of state control over industry. This reality may have motivated scholars to focus on these two countries. However, in general, the private equity field seems to have ignored other institutional contexts. In the future, authors should focus on the less explored institutional contexts of Africa, the Middle East, South Asia, and Latin America.

**Table 10:** Geographical focus of private equity research

	2001–2006	2007–2011	2012–2016	2017–2021	Total
**Panel A: Country share**					
Single country	34.48%	36.97%	37.74%	45.20%	40.17%
Multi country	22.41%	25.12%	37.11%	35.03%	32.73%
No geographical data reported	43.10%	37.91%	25.16%	19.77%	27.10%
**Panel B: Country**					
USA	45.00%	43.59%	56.67%	53.13%	51.85%
UK	5.00%	17.95%	11.67%	5.63%	10.05%
China	15.00%	3.85%	3.33%	13.75%	8.47%
Germany	5.00%	6.41%	1.67%	5.00%	4.23%
Italy	0.00%	3.85%	5.00%	3.13%	3.70%
Australia	10.00%	2.56%	4.17%	0.63%	2.65%
Netherlands	0.00%	3.85%	1.67%	1.88%	2.12%
Belgium	0.00%	2.56%	2.50%	0.63%	1.59%
France	0.00%	2.56%	0.83%	1.88%	1.59%
India	0.00%	5.13%	0.00%	1.25%	1.59%
Sweden	5.00%	0.00%	0.83%	1.88%	1.32%
South Africa	0.00%	2.56%	0.83%	1.25%	1.32%
Spain	0.00%	1.28%	0.83%	1.88%	1.32%
Japan	5.00%	1.28%	0.00%	1.88%	1.32%
Canada	10.00%	0.00%	1.67%	0.63%	1.32%
Taiwan	0.00%	0.00%	0.00%	1.88%	0.79%
Brazil	0.00%	0.00%	0.83%	1.25%	0.79%
Switzerland	0.00%	1.28%	0.00%	0.63%	0.53%
Norway	0.00%	0.00%	0.00%	1.25%	0.53%
South Korea	0.00%	0.00%	1.66%	0.00%	0.52%
Ireland	0.00%	0.00%	0.83%	0.00%	0.26%
Iceland	0.00%	0.00%	0.83%	0.00%	0.26%
Angola	0.00%	0.00%	0.83%	0.00%	0.26%
Tunisia	0.00%	0.00%	0.83%	0.00%	0.26%
Hungary	0.00%	0.00%	0.83%	0.00%	0.26%
Turkey	0.00%	0.00%	0.83%	0.00%	0.26%
Hong Kong	0.00%	0.00%	0.83%	0.00%	0.26%
Denmark	0.00%	1.28%	0.00%	0.00%	0.26%
New Zealand	0.00%	0.00%	0.00%	0.63%	0.26%

For our fifth research question (RQ5), we present an analysis of dominant methodologies that are employed in private equity research. We find that empirical methods have dominated the private equity field. Indeed, for research designs, scholars prefer quantitative methods (Table 11), and they also prominently use archival data. This is not surprising because research in the field primarily focuses on firm financing, which usually involves sourcing data and using empirical-quantitative research designs to establish causal relationships. It is important to note that other research designs have received some attention. However, more case studies and field studies can be conducted to grasp the mechanisms of private equity in a more real-world environment by focusing on the companies that employ them. Recent scholarship has also shown the advantage of using mixed methods in venture capital and private equity research (Levasseur et al., 2022). This will be useful for both finance researchers and students seeking to gain a better understanding of the subject.

**Table 11:** Methodological choice of private equity research

	**2001–2006**	**2007–2011**	**2012–2016**	**2017–2021**	**Total**
**Panel A: Research approach**					
Empirical	53.45%	57.35%	70.75%	75.14%	68.33%
Conceptual	15.52%	19.43%	14.47%	12.15%	14.77%
Modelling and analytical	6.90%	6.64%	5.66%	7.06%	6.48%
Review	1.72%	1.90%	1.57%	3.67%	2.44%
Mixed	0.00%	1.42%	0.31%	0.56%	0.64%
Not reported	22.41%	13.27%	7.23%	1.41%	7.33%
**Panel B: Research design**					
Quantitative	56.90%	55.45%	69.50%	75.42%	67.80%
Qualitative	18.97%	27.49%	20.44%	22.03%	22.53%
Mixed	0.00%	3.32%	2.52%	0.85%	1.91%
Not reported	24.14%	13.74%	7.55%	1.69%	7.76%
**Panel C: Research data**					
Archival	50.00%	52.61%	67.92%	74.01%	65.67%
Survey	5.17%	5.21%	5.03%	3.11%	4.36%
Case study	0.00%	3.32%	2.20%	2.26%	2.34%
Interview	1.72%	2.84%	1.89%	0.85%	1.70%
Experimental	0.00%	0.95%	0.63%	0.85%	0.74%
Field	0.00%	0.47%	0.63%	0.28%	0.43%
No data reported	43.10%	34.60%	21.70%	18.64%	24.76%

## *Science *mapping

### ***Co-authorship ***analysis

**Fig. 6:**
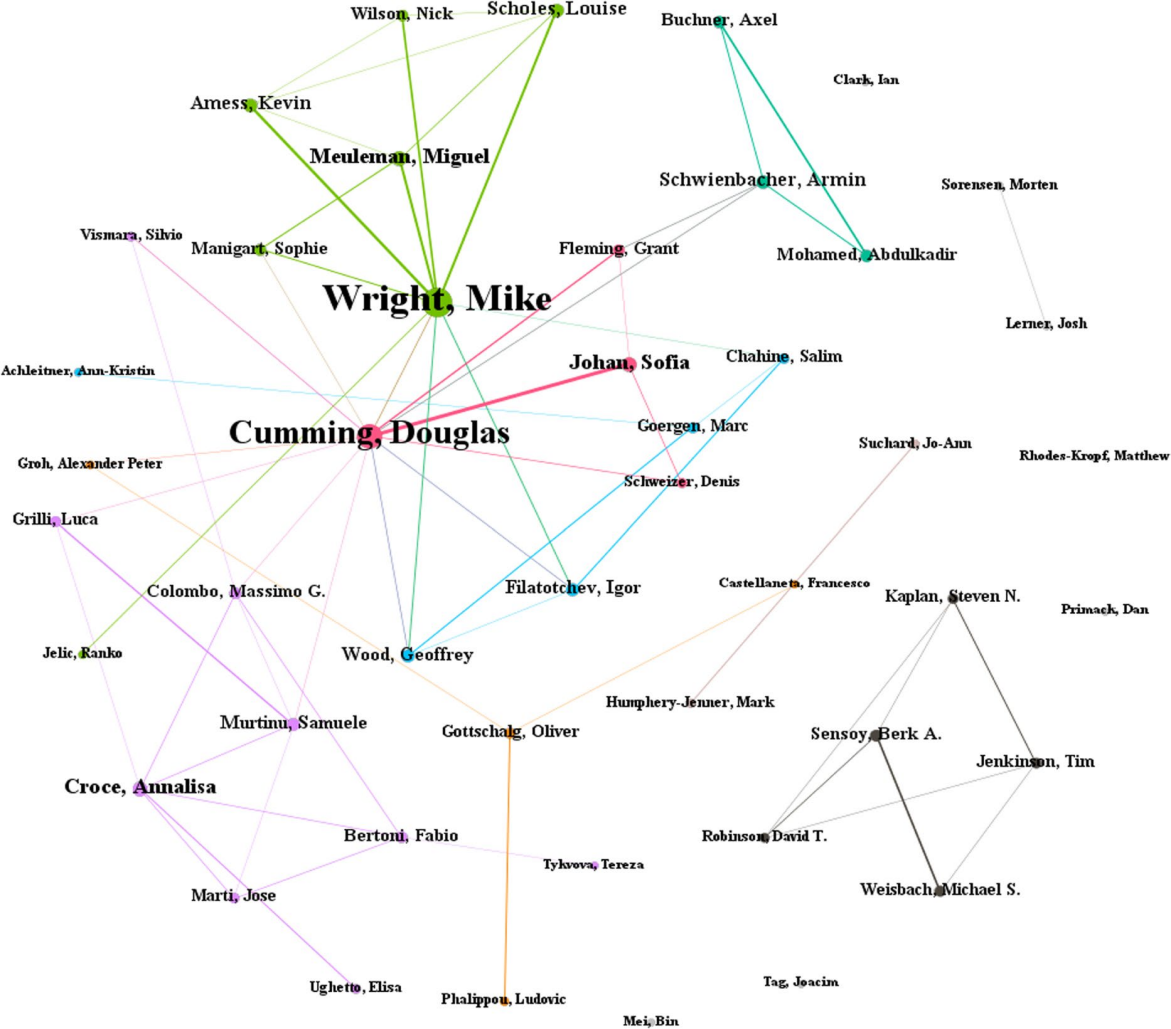
Co-authorship network of private equity researchers

To understand the research patterns in private equity research (RQ6), we present an analysis of co-authorship. This part of the analysis focuses on collaboration, with a particular focus on groups of authors who have produced works in the field. Since research is a collaborative endeavor, understanding the authors’ dynamics is important for learning how scholars conduct private equity research. We focused only on author groups with more than two authors which contain authors with 5 or more publications. Table 12 presents a summary of the author groups, while Fig. 6 presents the collaboration network.

**Table 12:** Summary of prominent author groups for private equity research

Author group	Author	Total link strength	Average publication year	Thematic focus	Geographical focus
#1	Annalisa Croce	12	2015.11	• Entrepreneurial finance	• Europe • Multi country
	Samuele Murtinu	9	2015.00		
	Fabio Bertoni	7	2015.86		
	Massimo G. Colombo	7	2015.00		
	Luca Grilli	6	2016.57		
	Jose Marti	5	2015.14		
	Elisa Ughetto	3	2015.67		
	Silvio Vismara	3	2017.30		
	Tereza Tykvova	1	2014.71		
#2	Mike Wright	38	2012.60	• Venture capital • Buyouts • Ownership and management	• Europe • UK
	Miguel Meuleman	12	2012.75		
	Kevin Amess	10	2013.43		
	Louise Scholes	10	2011.67		
	Sophie Manigart	7	2014.31		
	Nick Wilson	7	2015.20		
	Ranko Jelic	2	2015.20		
#3	Igor Filatotchev	9	2014.00	• Institutional aspects of private equity • Entrepreneurial finance	• Multi country
	Geoffrey Wood	9	2014.00		
	Salim Chahine	5	2010.50		
	Marc Goergen	5	2014.00		
	Ann-Kristin Achleitner	1	2012.00		
#4	Berk A. Sensoy	8	2014.55	• Venture capital firms • Private equity performance and valuation	• USA
	Michael S. Weisbach	6	2015.22		
	Tim Jenkinson	5	2017.67		
	Steven N. Kaplan	5	2014.90		
	David T. Robinson	4	2016.50		
#5	Oliver Gottschalg	5	2011.50	• Firm level outcomes of private equity • Buyouts	• Multi country • USA
	Ludovic Phalippou	3	2013.62		
	Alexander Peter Groh	2	2015.20		
	Francesco Castellaneta	1	2016.60		
#6	Douglas Cumming	30	2012.53	• Venture capital • Entrepreneurial finance • Corporate governance	• Multi country • Europe
	Sofia Johan	12	2014.07		
	Grant Fleming	7	2011.20		
	Denis Schweizer	4	2014.00		
#7	Armin Schwienbacher	10	2014.00	• Private equity investments	• USA
	Axel Buchner	8	2017.33		
	Abdulkadir Mohamed	8	2018.00

### Author group #1: Croce et al.

This is the largest group author group, and the author most central to the network is Annalisa Croce; hence, the cluster has been named after her. This is a set of scholars who have emerged more recently, as 2016 is the average year of first publication for all the authors in the group. Within the larger research area of private equity, the main theoretical focus of these authors is on entrepreneurial finance. Also, they occasionally discuss academia-based startups and high-tech entrepreneurial firms. In more recent times the group has focused on the alternative financing sources such as Initial Coin Offerings and Crowdfunding campaigns with authors such as Silvio Vismara being leading contributors to the field. In terms of geographical focus, this group is more focused on multi-country research, with a specific focus on Europe.

### Author group #2: Wright et al.

This is the second largest author group, with Mike Wright emerging as the most impactful author. In terms of the timing of their first publication, most of the authors in this group were first published around 2013, making their research slightly less recent than the first author group. Theoretically, this author group has mostly focused on venture capital and buyouts, ownership, and management in firms. These authors have also focused on publishing reviews and conceptual articles. The group’s empirical research focuses mostly on Europe—more specifically, the UK.

### Author group #3: Filatotchev et al.

The third author group is led by Igor Filatotchev. These authors’ first studies were also published around 2013, making them peers of the second author group. In terms of research themes, this author group is more focused on the institutional aspects of private equity and entrepreneurial finance. Geographically, the focus has been on multi-country studies.

### Author group #4: Sensoy et al.

This cluster’s average first publishing year is around 2016, meaning that the authors have published more recently. In terms of connectivity in the group, Berk A. Sensoy leads, and Michael Weisbach and Steven Kaplan are also in the group. Geographically, the authors focus more on the U.S. and are thematically oriented towards the workings of venture capital firms, as well as private equity performance and valuation.

### Author group #5: Gottschalg et al.

Oliver Gottschalg is the most connected author in this group; however, Ludovic Phalippou is the most prolific. Thematically, the group’s focus seems to be on firm-level outcomes of private equity and buyouts. Geographically, the focus is multi-country. However, in terms of single-country research, the group focuses on the USA.

### Author group #6: Cumming et al.

Douglas Cumming leads this author group. In terms of its size, the group is fairly small; however, it nevertheless generates a large number of papers relative to its larger counterparts. Cumming’s group also has strong connections to other groups. In terms of geographical focus, the author group focuses on international datasets, including but not limited to Europe. The thematic focus is similar to that of the first and second author groups, with a focus on venture capital, entrepreneurial finance, and corporate governance. The research choice is likely in part driven by the connection shared with these author groups.

### Author group #7: Schwienbacher et al.

In terms of the number of authors, this cluster is fairly small. Armin Schwienbacher leads the group in terms of connectivity, including connectivity with author group #6; however, the other two authors are not far behind in terms of network connectivity. This is another international-focused group, with a thematic focus on the nuances of private equity investments.

#### Co-citation analysis

We used co-citation analysis to answer the first part of our seventh and final research question. Based on the co-citation analysis of the cited literature, we arrived at four foundational themes. These themes represent the theoretical foundation of private equity research. To determine the number of articles to include in this analysis, we used local citation (i.e., the number of times that articles cited a reference within the corpus of articles in this study). This led to 189 articles, which, after the co-citation analysis, were divided into four document clusters, each representing a theme. The clusters were ordered based on the number of articles in each cluster. Table 13 presents the summary of the themes using co-citation analysis.

**Table 13:** Prominent themes in private equity research

Theme	Author(s)	Title	Source	Year	TC
**Theme #1: Venture capital** (TP: 89; TC: 3,490)					
**Most cited article**	Sahlman, WA	The structure and governance of venture-capital organizations	*Journal of Financial Economics*	1990	123
	Berger, AN; Udell, GF	The economics of small business finance: The roles of private equity and debt markets in the financial growth cycle	*Journal of Banking and Finance*	1998	98
	Hellmann, T; Puri, M	Venture capital and the professionalization of start-up firms: Empirical evidence	*Journal of Finance*	2002	85
	Kaplan, SN; Strömberg, P	Financial contracting theory meets the real world: An empirical analysis of venture capital contracts	*Review of Economic Studies*	2003	81
	Cumming, D; Walz, U	Private equity returns and disclosure around the world	*Journal of International Business Studies*	2010	77
**Key topics**	• Financial contracting • Financing lifecycle • Institutional aspects of venture capital				
**Theme #2: Buyouts and privatization** (TP: 45; TC: 1,937)					
**Most cited article**	Kaplan, SN; Strömberg, P	Leveraged buyouts and private equity	*Journal of Economic Perspectives*	2009	141
	Jensen, MC	Agency costs of free cash flow, corporate finance, and takeovers	*American Economic Review*	1986	120
	Kaplan, SN	The effects of management buyouts on operating performance and value	*Journal of Financial Economics*	1989	106
	Jensen, MC	Eclipse of the public corporation	*Harvard Business Review*	1989	84
	Cumming, D; Siegel, DS; Wright, M	Private equity, leveraged buyouts and governance	*Journal of Corporate Finance*	2007	83
**Key topics**	• Leveraged buyouts • Management buyouts • Agency cost of buyouts (management/owner conflict of interest)				
**Theme #3: Market mechanisms and venture capital backed IPOs** (TP: 29; TC: 1,044)					
**Most cited article**	Jensen, MC; Meckling, WH	Theory of the firm: Managerial behavior, agency costs and ownership structure	*Journal of Financial Economics*	1976	134
	Gompers, PA	Grandstanding in the venture capital industry	*Journal of Financial Economics*	1996	75
	Myers, SC; Majluf, NS	Corporate financing and investment decisions when firms have information that investors do not have	*Journal of Financial Economics*	1984	64
	Heckman, JJ	Sample selection bias as a specification error	*Econometrica*	1979	54
	Barry, CB; Muscarella, CJ; Peavy III, JW; Vetsuypens, MR	The role of venture capital in the creation of public companies. Evidence from the going-public process	*Journal of Financial Economics*	1990	43
**Key topics**	• Market mechanisms • Information asymmetry Effect of venture capital on firm’s decision to go public				
**Theme #4: Valuation and performance of private equity investment** (TP: 26; TC: 1,320)					
**Most cited article**	Kaplan, SN; Schoar, A	Private equity performance: Returns, persistence, and capital flows	*Journal of Finance*	2005	205
	Metrick, A; Yasuda, A	The economics of private equity funds	*Review of Financial Studies*	2010	91
	Phalippou, L; Gottschalg, O	The performance of private equity funds	*Review of Financial Studies*	2009	90
	Cochrane, JH	The risk and return of venture capital	*Journal of Financial Economics*	2005	84
	Gompers, P; Lerner, J	Money chasing deals? The impact of fund inflows on private equity valuations	*Journal of Financial Economics*	2000	83
**Key topics**	Factors affecting performance and valuation of private equity investments				

*TP* total publications. *TC* total citations received from publications within the dataset used in this study

Note that co-citation analysis gives rise to papers in Table 13 that are not private equity papers, such as Heckman’s and Myers and Majluf’s works. Those papers just happen to be papers that are most often cited in private equity papers.

### Theme #1: Venture capital

This is the largest foundational theme, containing 89 literature references that focus on *venture capital*. Specifically, the theme here relates to venture capital and the performance of venture capital investments. The fact that venture capital is the largest foundational theme indicates its importance—especially because authors cover it from different aspects. This is unsurprising, as venture capital is one of the most popular methods by which businesses can receive private equity. Sahlman (1990) studies the structures of venture capital firms and their relationship with leveraged buyouts. Berger and Udell (1998) study the role of venture capital in the financial growth cycle of small firms. Other research under this theme focuses on the role of venture capital in startups (Hellmann & Puri, 2002), financial contracting (Admati & Pfleiderer, 1994; Cumming & Johan, 2013; Kaplan & Stromberg, 2003), venture capital cycle performance (Hochberg et al., 2007), capital market structures (Black & Gilson, 1998), disclosures by venture capital firms (D. Cumming & Walz, 2010; Johan & Zhang, 2021a), and control and oversight by venture capital (P. A. Gompers, 1995; Lerner, 1995). The scholars in this area have also focused on the various aspects of venture capital, such as the outcomes of venture capital (i.e., investment performance), the process of venture capital (i.e., monitoring and contracting), the avenues of investment for venture capital (i.e., small businesses and startups), and institutional factors (i.e., capital market structure and law and economic systems). Primarily, the foundations of private equity research have focused on venture capital. Future research should focus on other sources of private equity, such as angel investors or crowdfunding (Vismara, 2016). While more recent research may extend to other sources of private equity, the foundations will remain in venture capital.

The overlap between venture capital and private equity explains a large part of this theme. Many private equity funds style drift into venture capital deals, and vice versa (Koenig & Burghof, 2022). For example, Cumming, Fleming, and Schwienbacher (2009) report that 35.5% of early-stage venture capital deals are done by late-stage private equity funds. And many research papers in the area of private equity comingle data on venture capital and private equity due to the frequent style drift and similarity in transactions and issues that arise.

### Theme #2: Buyouts and privatization

The second foundational theme in private equity research is the process of *privatization*—more specifically, *buyouts*. Buyouts are a primary method of privatization, through which a firm can switch from a public corporation to private equity. The focus in the field has been on leveraged buyouts and management buyouts. The former contends with the acquisition of shares using borrowed funds (S. N. Kaplan & Strömberg, 2009), and the latter is about management buying shares of the companies it manages. The literature also points to the prevalence of agency theory in the literature, which theoretically focuses on the role of management. Some scholars predict that such takeovers are indicative of conflicts of interest between management and shareholders (Jensen, 1986). However, management buyouts have also been found to be associated with increased operational efficiency (S. Kaplan, 1989), and increased managerial discretion can benefit firm growth (Wright et al., 2000). The debate over the role of management thus forms one of the foundational topics in the research on buyouts. Other important topics are the outcomes of such buyouts and their effect on governance (D. Cumming et al., 2007) and productivity and efficiency (Harris et al., 2005; Lichtenberg & Siegel, 1990). While earlier research shows innovation improvements with private equity deals (Lerner et al., 2011), more recent work shows the exact opposite using the same empirical methods with more recent data (Cummings et al., 2020). In addition to the major topics discussed above, the other major topic is the process of buyouts. In this subarea, scholars focus on how leveraged buyouts are financed (Demiroglu & James, 2010) and what determines buyout activities (Opler & Titman, 1993).

### Theme #3: Market mechanisms and venture capital-backed IPOs

The third foundational theme in private equity research focuses on *market mechanisms and venture capital-backed IPOs*. The discussions regarding market forces (Akerlof, 1970)—especially information asymmetry (Leland & Pyle, 1977; Myers & Majluf, 1984)—have been prevalent under this theme. It is noteworthy that this theme is about public corporations. More specifically, the cluster centers on the decision to go public and the role that different market forces play in this decision. Under this theme, researchers have discussed various topics relating to the nature of venture capital firms (P. A. Gompers, 1996) and their effect on a firm’s decision to go public (Barry et al., 1990; P. M. Lee & Wahal, 2004; Lerner, 1994). Importantly, researchers discuss the role of private equity on IPOs’ performance (Bruton et al., 2010). This sort of research represents an older stream of finance literature, which focused on IPOs while also presenting the roots of private equity research. In this stream of research, the roots of private equity research stemmed from research in public corporations and IPOs. As the previous two clusters show, the direction of research has shifted more towards privatization. This, in turn, presents an interesting insight into how the private equity field has developed over time.

### Theme #4: Valuation and performance of private equity investments

Though this foundational theme is minor due to its relatively smaller size, it is also very interesting, as the average year of publication for the cited articles is around 2008. For context, the average publication years for the first three themes are 2001, 2002, and 1991, respectively. Thus, this foundational theme—while still important—developed more recently than the others. The discussions in the theme have revolved around the *valuation and performance of private equity investments*. Kaplan and Schoar’s (2005) study finds that the return on private equity investments grows differently from the one in mutual funds; indeed, the growth in the funds is contingent on the performance of the private equity partnership and the size of the fund. Metrick and Yasuda (2010) report that performance differs across types of funds, with buyout funds outperforming venture capital funds. Cumming and Walz (2010) first established (dating back to 2004 when the paper was first released as a working paper) that private equity funds misreport valuations to institutional investors, and those mis-valuations are correlated with proxies for information asymmetry based on national-level institutions and firm-specific and deal-specific characteristics. Smith et al. (2022) show that in the USA, the Freedom of Information Act plays a disciplinary role in mitigating private equity fund mis-valuations. Phalippou and Gottschalg (2009) find that the performance results are often biased towards the better performing funds, and funds’ underperformance increased when the researchers took risk into account. The results obtained by Gompers and Lerner (2000) show that the capital inflows into venture capital firms increase the valuation of their investments. The research in this theme has therefore focused on the performance of private equity funds, including the characteristics of private equity investment and whether private equity firms are determinants of performance.

#### Emergent research frontiers in private equity research

To answer the second part of our seventh and final research question (RQ7), Table 14 presents the summary of emergent research frontiers, along with potential future directions for research. In this section, we present the analysis of the articles published within the last three years at the time of writing (i.e., between 2019 and 2021). We used bibliographic coupling to create thematic clusters, each of which represents a research frontier on which authors have recently focused. These frontiers can be further developed. In the following discussion, we present an analysis of these research frontiers and suggest areas for future research.

**Table 14:** Emergent research frontiers in private equity research

Frontier	Author	Title	Year	Source	TC
**Frontier #1: Private equity and strategy** (TP: 63, TC: 116)					
**Most cited article**	Villalonga, B; Amit, R	Family ownership	2020	*Oxford Review of Economic Policy*	9
	Bernstein, S; Lerner, J; Mezzanotti, F	Private equity and financial fragility during the crisis	2019	*Review of Financial Studies*	9
	Jelic, R; Zhou, D; Wright, M	Sustaining the buyout governance model: Inside secondary management buyout boards	2019	*British Journal of Management*	8
	Toms, S; Wilson, N; Wright, M	Innovation, intermediation, and the nature of entrepreneurship: A historical perspective	2020	*Strategic Entrepreneurship Journal*	7
	Gentry, M; Stroup, C	Entry and competition in takeover auctions	2019	*Journal of Financial Economics*	6
**Future research**	• Is private equity a superior form of ownership compared to public corporation? • What is the effect of exogenous shocks on private equity investments and financing choices? • What role does private equity play in the cycles and waves of privatization?				
**Frontier #2**: **Alternative financing and firm outcomes** (TP: 55, TC: 284)					
**Most cited article**	Huang, W; Meoli, M; Vismara, S	The geography of initial coin offerings	2020	*Small Business Economics*	31
	Cummings, ME; Rawhouse, H; Vismara, S; Hamilton, EL	An equity crowdfunding research agenda: Evidence from stakeholder participation in the rulemaking process	2020	*Small Business Economics*	23
	Cumming, D; Deloof, M; Manigart, S; Wright, M	New directions in entrepreneurial finance	2019	*Journal of Banking and Finance*	21
	Fisch, C; Momtaz, PP	Institutional investors and post-ICO performance: An empirical analysis of investor returns in initial coin offerings (ICOs)	2020	*Journal of Corporate Finance*	18
	Bertoni, F; Marti, J; Reverte, C	The impact of government-supported participative loans on the growth of entrepreneurial ventures	2019	*Research Policy*	17
**Future research**	• What are the enablers, barriers, and consequences of alternative financing methods, including their similarities and differences? • How are the different forms of alternative financing governed, what are their similarities and differences, and how should firms go about managing the governance for difference sources of finance if they choose to pursue a diversified financing strategy predicated on alternative finance? • What are the factors driving entrepreneurial staging and manipulation across alternative financing methods and how can such manipulation be identified and mitigated?				
**Frontier #3: Private equity investment outcomes** (TP: 44, TC: 119)					
**Most cited article**	Brown, GW; Gredil, OR; Kaplan, SN	Do private equity funds manipulate reported returns?	2019	*Journal of Financial Economics*	19
	Barber, BM; Morse, A; Yasuda, A	Impact investing	2021	*Journal of Financial Economics*	16
	Nadauld, TD; Sensoy, BA; Vorkink, K; Weisbach, MS	The liquidity cost of private equity investments: Evidence from secondary market transactions	2019	*Journal of Financial Economics*	10
	Ennis, RM	Institutional investment strategy and manager choice: A critique	2020	*Journal of Portfolio Management*	9
	Platanakis, E; Sakkas, A; Sutcliffe, C	Harmful diversification: Evidence from alternative investments	2019	*British Accounting Review*	7
**Future research**	• What are the factors affecting the returns and valuations on private equity investments? • How does impact investing affects the economy and sustainable development? • What are the factors driving investing behavior of private equity investors across generations in an increasingly disruptive, volatile, uncertain, complex, and ambiguous environment?				
**Frontier #4: Private equity and entrepreneurship** (TP: 27, TC: 123)					
**Most cited article**	Bertoni, F; Colombo, MG; Quas, A	The role of governmental venture capital in the venture capital ecosystem: An organizational ecology perspective	2019	*Entrepreneurship Theory and Practice*	21
	Giraudo, E; Giudici, G; Grilli, L	Entrepreneurship policy and the financing of young innovative companies: Evidence from the Italian Startup Act	2019	*Research Policy*	15
	Sun, SL; Chen, VZ; Sunny, SA; Chen, J	Venture capital as an innovation ecosystem engineer in an emerging market	2019	*International Business Review*	15
	Vanacker, T; Forbes, DP; Knockaert, M; Manigart, S	Signal strength, media attention, and resource mobilization: Evidence from new private equity firms	2020	*Academy of Management Journal*	15
	Shinkle, GA; Suchard, JA	Innovation in newly public firms: The influence of government grants, venture capital, and private equity	2019	*Australian Journal of Management*	11
**Future research**	• How does private equity help entrepreneurs across contexts and industries gain access to resources other than financial ones? • How do entrepreneur preference and outlook of private equity change over time across contexts and industries? • How can private equity remain relevant in tandem with how entrepreneurial ventures evolve over time across contexts and industries?				

*TP* total publications. *TC* total citations received from publications within the dataset used in this study

### Frontier #1: Private equity and strategy

*Private equity and strategy* represent the largest emergent research frontier in recent years. Though researchers discuss a range of topics in this front, such as ownership, mergers and acquisitions, and corporate governance, the primary focus in this cluster is on strategic management and its relationship with private equity. In this context, studies have focused on the role of private equity firms as monitors, as well as their role in the firms and economy at large (Aldatmaz & Brown, 2020). Bernstein et al. (2019) discuss private equity investments during financial crises, while Jelic et al. (2019) focus on the effects of private equity on management buyouts. Researchers also discuss other strategic decisions, such as takeover auctions (Gentry & Stroup, 2019) and hedge fund activism (Buchanan et al., 2020). More recently, the focus has been on human resource management—especially in the context of the COVID-19 pandemic (Collings et al., 2021) and workplace safety (Cohn et al., 2021). Block et al., (2019a, 2019b) present several research criteria for investors. The posit that major criteria in PE investment include revenue growth, value-added of product/service, and management team track record, while international scalability, current profitability, business model, and the reputation of existing investors are considered of lower importance. The discussions have tended to focus on the effect that investors have on firm performance and value, with many studies focusing on the non-financial outcomes of industries such as education (Eaton et al., 2020) and nursing homes (S. S. Huang & Bowblis, 2019). The growth in private equity seems to follow the predictions of Michael Jensen, who had predicted that private equity would eclipse public corporations because it is a superior form of ownership (Morris & Phalippou, 2020). The growing importance of private equity seems to confirm this prediction, but more work has to be done to evaluate the “quality” aspect of private equity. This has led to more scholars studying the strategic aspects of private equity. In addition, scholars also need to focus on the recent pandemic to analyze changes in firms’ financing choices. Moreover, we do not discount the possibility that the privatization of public corporations is not a linear affair, as firms strategize for survival and success. In this regard, it may be worthwhile for future research to examine the cycles and waves of privatization strategy and the role of private equity in respective phases of that strategy. Thus, future research can consider pursuing the following research questions:

- *Is private equity a superior form of ownership compared to public corporation?*
- *What is the effect of exogenous shocks on private equity investments and financing choices?*
- *What role does private equity play in the cycles and waves of privatization?*

### Frontier #2: Alternative financing and firm outcomes

The second emergent research frontier focuses on *alternative financing and firm outcomes*, with a particular focus on sources such as crowdfunding and angel investors. While venture capital firms remain an important source of private equity, alternative financing avenues have recently gained prominence. The exploration of new directions of entrepreneurial finance has been the primary motivator for such research. The topics explored include initial coin offerings (ICO) (Fisch & Momtaz, 2020; W. Huang et al., 2020; Meoli & Vismara, 2022), equity crowdfunding (Cummings et al., 2020), government-supported participative loans (Bertoni, Martí, et al., 2019a, 2019b), and angel investment (D. Cumming & Zhang, 2019). Both the exploration of entrepreneurial finance methods and their relationship with firm performance have garnered much attention (Vismara, 2022). Fisch and Momtaz (2020) focus on post-ICO performance, finding that institutional investor backing is associated with a high ICO performance. Cirillo et al. (2019) focus on research and development in family firms and the role that private equity and banks play in these firms. Yung (2019) explores entrepreneurial manipulation and staged financing. Bongini et al. (2021) explore market-based financing and SME access. Finally, Collewaert et al. (2021) study angel investors’ post-investment governance. In this research front, more focus has been given to alternate sources of private equity. This is all the more important as many such offerings may not result in monetary gains for the investors (Signori & Vismara, 2018). Hence, one possible avenue for future research would be to compare and contrast the enablers, barriers, and consequences of alternative financing methods. Moreover, the plethora of alternative financing methods also points to the need to understand its governance, as well as possible manipulation that may occur so as to safeguard both the investors participating in and the firms receiving funding from alternative financing. Thus, future research may pursue the following research questions:

- *What are the enablers, barriers, and consequences of alternative financing methods, including their similarities and differences?*
- *How are the different forms of alternative financing governed, what are their similarities and differences, and how should firms go about managing the governance for different sources of finance if they choose to pursue a diversified financing strategy predicated on alternative finance?*
- *What are the factors driving entrepreneurial staging and manipulation across alternative financing methods and how can such manipulation be identified and mitigated?*

### Frontier #3: Private equity investment outcomes

The third emergent research frontier deals with *private equity investment outcomes*. Unlike the second emergent research frontier, which focuses on firm outcomes and their association with private equity, this cluster is more concerned with private equity investment outcomes. Researchers’ interests relate to different aspects of private equity investments, including manipulated returns of private equity investments (Brown et al., 2019a, 2019b; Cumming & Walz, 2010), impact investing (Barber et al., 2021), investment costs (Nadauld et al., 2019), diversification and portfolio (Delfim & Hoesli, 2019; Platanakis et al., 2019), risk (Arnold et al., 2019), and the role of education ties in driving returns (Fuchs et al., 2022). In addition, researchers have explored the investor side of private equity by looking at the various types of investments and exploring private equity investors’ investment behavior. Andonov et al. (2021) explore the performance of private and public institutional investors in the infrastructure sector, finding that private institutional investors perform better than public ones. Batt and Appelbaum (2021) look at private equity from a corporate governance perspective. In the future, researchers may wish to explore the nuances of private equity investments by focusing on their returns and valuations, as well as the effects of impact investing. In addition, future research should (re)explore and (re)update our understanding of investor behavior due to the constant changes emerging from new and transitioned generations, as well as the new reality of an increasingly disruptive, volatile, uncertain, complex, and ambiguous (DVUCA) environment. Thus, future research is encouraged to consider the following research questions:

- *What are the factors affecting the returns and valuations on private equity investments?*
- *How does impact investing affect the economy and sustainable development?*
- *What are the factors driving investing behavior of private equity investors across generations in an increasingly disruptive, volatile, uncertain, complex, and ambiguous environment?*
- *To what extent are private equity investment decisions and outcomes associated with fraud risk and actual fraud?*

### Frontier #4: Private equity and entrepreneurship

The final emergent research frontier deals with the role of *private equity in entrepreneurship*. The most cited article under this theme, by Bertoni et al. (2019a), focuses on the role of government venture capital in the development of the entrepreneurial ecosystem. In a similar vein, Giraudo et al. (2019) explore the role of entrepreneurship policy. The discussion here suggests a focus on institutional factors and their impact on the entrepreneurial ecosystem. Other research has focused on the role that private equity firms play in firm competencies (e.g., innovation) (Sun et al., 2019) and firm quality (Vanacker et al., 2020). Apart from the research streams mentioned above, researchers have also focused on the life cycle of venture capital firms (Ma, 2020). More recently, research has focused on the effect of private equity investment on the acquisition of non-financial resources (Quas et al., 2021) such as research and development (R&D) (Chahine et al., 2021a, 2021b). Hence, research in this frontier has delved into the non-financial gains of private equity investments in entrepreneurship. Entrepreneurial finance has been a major focus of research in recent times; therefore, it makes sense to further explore the topic by investigating how private equity helps entrepreneurship gain access to resources other than financial ones. Moreover, as entrepreneurs grow over time, their preference and outlook of private equity may also change. Similarly, as entrepreneurial ventures transition from startups to scaleups, the need for private equity will also evolve. In this regard, future researchers should not view entrepreneurial finance as a fixed state, but rather as a dynamic, evolutionary phenomenon in private equity research. Moreover, it is important to remember that not entrepreneurial ventures are the same, and thus, future research will need to account for the unique peculiarities in entrepreneurship across contexts (e.g., digital versus brick-and-mortar retail, developed versus developing country, small versus medium enterprises) and industries (e.g., manufacturing versus services). Thus, future research aligned to the following research questions are likely to be potentially fruitful:

- *How does private equity help entrepreneurs across contexts and industries gain access to resources other than financial ones?*
- *How do entrepreneur preference and outlook on private equity change over time across contexts and industries?*
- *How can private equity remain relevant in tandem with how entrepreneurial ventures evolve over time across contexts and industries?*
- *How does private equity affect the real outcomes of the firms in which it finances, including non-financial performance metrics?*

## Conclusion

This study used bibliometric analysis to present a comprehensive encapsulation of the fields of venture capital and private equity. We aimed to achieve three research objectives by using a range of tools, including performance analysis, co-authorship analysis, co-citation analysis, and bibliographic coupling.

The data over the sample period 2001 to 2021 indicate that a number of prominent scholars have contributed to research in both fields. In the methodology analysis, we found that the number of studies on single-country and multi-country research was almost equal for private equity while venture capital studies trend in favor of a single country. Further, our analysis also showed that, in single-country studies, researchers have focused mostly on the USA in both venture capital and private equity; more recently, however, researchers have also explored the Chinese context. The institutional contexts of less researched areas, such as Africa, the Middle East, South Asia, and Latin America, present opportunities for future research.

The thematic analysis of venture capital research revealed that the foundational themes in the field include *venture capital adoption and financing processes*, *venture capital roles in business*, *venture capital governance*, *venture capital syndication*, and *venture capital and creation of public organizations*, whereas the field’s emergent themes or frontiers in recent times include *venture capital and sustainable entrepreneurship*, *fintech and crowdfunding*, *venture capital investment strategies*, *venture capital and innovation*, *entrepreneurial finance*, *venture capital and IPOs*, and *drivers of venture capital funding decisions*. Whereas the thematic analysis of private equity research showed that *venture capital* is one of the field’s foundational themes alongside *buyouts and privatization*, *market mechanisms and venture capital backed IPOs*, and *performance and valuation of private equity investments*. In more recent times, authors have focused on *private equity and strategy*, *alternative financing and firm outcomes*, *private equity investment outcomes*, and *private equity and entrepreneurship*. Indeed, research in venture capital and private equity, though still rooted in financing research on capital budgeting and IPO, has grown to include a range of topics, with entrepreneurial finance and behavioral aspects of venture capital and private equity investments being the most prominent ones. In the future, scholars should further explore these areas to advance these fields.

## Figures and Tables

**Fig. 7:**
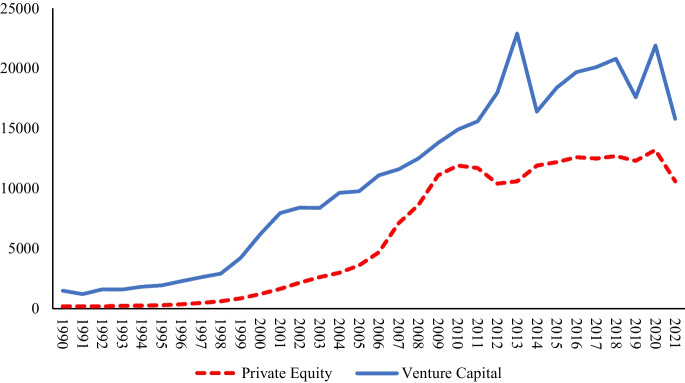
Documents with the words “venture capital” and “private equity” found on Google Scholar

## Appendix

This appendix presents Google Scholar data dating back to 1990 on documents relating to venture capital and private equity.

Figure 7 shows a significant growth in venture capital and private equity research starting in the late 1990s and early 2000s. The Google Scholar hits are to both published and working papers. Some early scholars that worked on venture capital and private equity starting in the early 1990s include Mike Wright (106,150 citations on Google Scholar as of January 15, 2022), Josh Lerner (63,781 citations on Google Scholar as of January 15, 2022), and Paul Gompers (45,715 citations on Google Scholar as of January 15, 2022).

In contrast, the data in the present study comprised of published papers only for the period of 2001 to 2021. The period was originally selected by the second author and then agreed upon by all authors, to cover a period over which there was a rapid expansion of venture capital and private equity research and a wide breadth of research on topic. The data for earlier periods showed a much smaller number of authors and topic areas within these fields, and hence a systematic analysis and review of those earlier periods are not a part of this study. Also, the more remote data offer fewer insights into current trends and future research directions, which were the main aims of this study. The time period was not selected for any particular reverse engineering of results.

It is also interesting that the Google Scholar data shows a leveling off for private equity research around 2009, and venture capital around 2013. These trends could be explained by the maturing of both fields, with many foundational insights already researched and reported at this stage. Also, these trends might be explained by the emergence and massive growth of crowdfunding and fintech and research, which are two areas that many venture capital and private equity scholars might have migrated to since the early 2010s. Future research could analyze and explain these trends.

## References

[CR1] Acedo, F. J., Barroso, C., Casanueva, C., & Galan, J. L. (2006). Co-authorship in management and organizational studies: An empirical and network analysis. *Journal of Management Studies,**43*(5), 957–983. 10.1111/j.1467-6486.2006.00625.x

[CR2] Admati, A. R., & Pfleiderer, P. (1994). Robust financial contracting and the role of venture capitalists. *The Journal of Finance,**49*(2), 371–402. 10.1111/j.1540-6261.1994.tb05146.x

[CR3] Ahluwalia, S., Mahto, R. V., & Guerrero, M. (2020). Blockchain technology and startup financing: A transaction cost economics perspective. *Technological Forecasting and Social Change*, *151*(November 2019), 119854. 10.1016/j.techfore.2019.119854

[CR4] Akerlof, G. A. (1970). The market for “lemons”: Quality uncertainty and the market mechanism. *The Quarterly Journal of Economics,**84*(3), 488. 10.2307/1879431

[CR5] Aldatmaz, S., & Brown, G. W. (2020). Private equity in the global economy: Evidence on industry spillovers. *Journal of Corporate Finance,**60*, 101524. 10.1016/j.jcorpfin.2019.101524

[CR6] Amornsiripanitch, N., Gompers, P. A., & Xuan, Y. (2019). More than money: Venture capitalists on boards. *Journal of Law, Economics, and Organization,**35*(3), 513–543. 10.1093/jleo/ewz010

[CR7] Andersen, N. (2019). Mapping the expatriate literature: A bibliometric review of the field from 1998 to 2017 and identification of current research fronts. *International Journal of Human Resource Management*, *0*(0), 1–38.10.1080/09585192.2019.1661267

[CR8] Andonov, A., Kräussl, R., & Rauh, J. (2021). Institutional investors and infrastructure investing. *Review of Financial Studies,**34*(8), 3880–3934. 10.1093/rfs/hhab048

[CR9] Arnold, T. R., Ling, D. C., & Naranjo, A. (2019). Private equity real estate funds: Returns, riskexposures, and persistence. *Journal of Portfolio Management,**45*(7), 24–42. 10.3905/jpm.2019.1.103

[CR10] Babich, V., Marinesi, S., & Tsoukalas, G. (2020). Does crowdfunding benefit entrepreneurs and venture capital investors? *Manufacturing & Service Operations Management*, (July), msom.2019.0835. 10.1287/msom.2019.0835

[CR11] Baker, H. K., Kumar, S., & Pandey, N. (2020). A bibliometric analysis of European Financial Management ʼs first 25 years. *European Financial Management,**26*(5), 1224–1260. 10.1111/eufm.12286

[CR12] Barber, B. M., Morse, A., & Yasuda, A. (2021). Impact investing. *Journal of Financial Economics,**139*(1), 162–185. 10.1016/j.jfineco.2020.07.008

[CR13] Barney, J. (1991). Firm resources and sustained competitive advantage. *Journal of Management,**17*(1), 99–120. 10.1177/014920639101700108

[CR14] Barry, C. B., Muscarella, C. J., Peavy, J. W., & Vetsuypens, M. R. (1990). The role of venture capital in the creation of public companies. Evidence from the going-public process. *Journal of Financial Economics*, *27*(2), 447–471. 10.1016/0304-405X(90)90064-7

[CR15] Bastian, M., Heymann, S., & Jacomy, M. (2009). Gephi: An open source software for exploring and manipulating networks. In *Proceedings of the Third International ICWSM Conference*, 361–362. 10.1136/qshc.2004.010033

[CR16] Batt, R., & Appelbaum, E. (2021). *Private equity at work: When wall street manages main street*. Russell Sage Foundation Press.

[CR17] Baum, J. A. C., & Silverman, B. S. (2004). Picking winners or building them? Alliance, intellectual, and human capital as selection criteria in venture financing and performance of biotechnology startups. *Journal of Business Venturing,**19*(3), 411–436. 10.1016/S0883-9026(03)00038-7

[CR18] Berger, A. N., & Udell, G. F. (1998). The economics of small business finance: The roles of private equity and debt markets in the financial growth cycle. *Journal of Banking & Finance,**22*(6–8), 613–673. 10.1016/S0378-4266(98)00038-7

[CR19] Bernstein, S., Lerner, J., & Mezzanotti, F. (2019). Private equity and financial fragility during the crisis. *Review of Financial Studies,**32*(4), 1309–1373. 10.1093/rfs/hhy078

[CR20] Bertoni, F., Martí, J., & Reverte, C. (2019). The impact of government-supported participative loans on the growth of entrepreneurial ventures. *Research Policy,**48*(1), 371–384. 10.1016/j.respol.2018.09.006

[CR21] Bertoni, F., Colombo, M. G., & Quas, A. (2019a). The role of governmental venture capital in the venture capital ecosystem: An organizational ecology perspective. *Entrepreneurship: Theory and Practice*, *43*(3), 611–628. 10.1177/1042258717735303

[CR22] Bianchini, R., & Croce, A. (2022). The role of environmental policies in promoting venture capital investments in cleantech companies. *Review Corporate Finance* (Forthcoming).

[CR23] Black, B. S., & Gilson, R. J. (1998). Venture capital and the structure of capital markets: Banks versus stock markets. *Journal of Financial Economics,**47*(3), 243–277. 10.1016/S0304-405X(97)00045-7

[CR24] Block, J., Colombo, M., Cumming, D., & Vismara, S. (2018). New players in entrepreneurial finance and why they are there. *Small Business Economics,**50*, 239–250. 10.1007/s11187-016-9826-6

[CR25] Block, J., Fisch, C., Vismara, S., & Andres, R. (2019). Private equity investment criteria: An experimental conjoint analysis of venture capital, business angels, and family offices. *Journal of Corporate Finance,**58*(March), 329–352. 10.1016/j.jcorpfin.2019.05.009

[CR26] Block, J. H., Fisch, C. O., Obschonka, M., & Sandner, P. G. (2019). A personality perspective on business angel syndication ✰. *Journal of Banking and Finance,**100*, 306–327. 10.1016/j.jbankfin.2018.10.006

[CR27] Block, J. H., Groh, A., Hornuf, L., Vanacker, T., & Vismara, S. (2021). The entrepreneurial finance markets of the future: A comparison of crowdfunding and initial coin offerings. *Small Business Economics,**57*(2), 865–882. 10.1007/s11187-020-00330-2

[CR28] Bongini, P., Ferrando, A., Rossi, E., & Rossolini, M. (2021). SME access to market-based finance across Eurozone countries. *Small Business Economics,**56*(4), 1667–1697. 10.1007/s11187-019-00285-z

[CR29] Brander, J. A., Amit, R., & Antweiler, W. (2002). Venture Selection vs. the Value-Added Hypothesis. *Journal of Economics & Management Strategy*, *11*(3), 423–452.

[CR30] Brav, O. (2009). Access to capital, capital structure, and the funding of the firm. *Journal of Finance,**64*(1), 263–308. 10.1111/j.1540-6261.2008.01434.x

[CR31] Brown, G. W., Gredil, O. R., & Kaplan, S. N. (2019). Do private equity funds manipulate reported returns? *Journal of Financial Economics,**132*(2), 267–297. 10.1016/j.jfineco.2018.10.011

[CR32] Brown, R., Mawson, S., & Rowe, A. (2019b). Start-ups, entrepreneurial networks and equity crowdfunding: A processual perspective. *Industrial Marketing Management*, *80*(February 2018), 115–125. 10.1016/j.indmarman.2018.02.003

[CR33] Budhwar, P., Cumming, D., & Wood, G. (2022). Editorial: Entrepreneurial finance and the legacy of Mike Wright. *British Journal of Management, 33*(1), 3–8. 10.1111/1467-8551.12535

[CR34] Bruton, G. D., Filatotchev, I., Chahine, S., & Wright, M. (2010). Governance, ownership structure, and performance of IPO firms: The impact of different types of private equity investors and institutional environments. *Strategic Management Journal,**31*(5), 491–509. 10.1002/smj.822

[CR35] Buchanan, J., Chai, D. H., & Deakin, S. (2020). Unexpected corporate outcomes from hedge fund activism in Japan. *Socio-Economic Review,**18*(1), 31–52. 10.1093/ser/mwy007

[CR36] Cumming, D. J., & Johan, S. A. (2017). The problems with and promise of entrepreneurial finance. *Strategic Entrepreneruship Journal, 11*(3), 357–370. 10.1002/sej.1265

[CR37] Butticè, V., & Vismara, S. (2022). Inclusive digital finance: The industry of equity crowdfunding. *Journal of Technology Transfer, Forthcoming*. 10.1007/s10961-021-09875-0

[CR38] Bygrave, W. D. (1987). Syndicated investments by venture capital firms: A networking perspective. *Journal of Business Venturing,**2*(2), 139–154. 10.1016/0883-9026(87)90004-8

[CR39] Cavallo, A., Ghezzi, A., Dell’Era, C., & Pellizzoni, E. (2019). Fostering digital entrepreneurship from startup to scaleup: The role of venture capital funds and angel groups. *Technological Forecasting and Social Change,**145*(April), 24–35. 10.1016/j.techfore.2019.04.022

[CR40] Chabowski, B. R., Saimee, S., & Hult, G. T. M. (2013). A bibliometric analysis of the global research on sofosbuvir. *Journal of internatinal business studies*, *44*(1), 234–622. 10.12688/f1000research.12314.1

[CR41] Chahine, S., Filatotchev, I., Bruton, G. D., & Wright, M. (2021). Success by Association”: The Impact of Venture Capital Firm Reputation Trend on Initial Public Offering Valuations. *Journal of Management,**47*(2), 368–398. 10.1177/0149206319847265

[CR42] Chahine, S., Saade, S., & Goergen, M. (2019). Foreign business activities, foreignness of the VC syndicate, and IPO value. *Entrepreneurship: Theory and Practice*, *43*(5), 947–973. 10.1177/1042258718757503

[CR43] Chahine, S., Goergen, M., & Saade, S. (2021b). Foreign venture capitalists and access to foreign research: The case of US initial public offerings. *British Journal of Management*, *0*, 1–21. 10.1111/1467-8551.12451

[CR44] Cheng, C. Y., & Tang, M. J. (2019). Partner-selection effects on venture capital investment performance with uncertainties. *Journal of Business Research*, *95*(June 2017), 242–252. 10.1016/j.jbusres.2018.10.002

[CR45] Chowdhury, F., Audretsch, D. B., & Belitski, M. (2019). Institutions and entrepreneurship quality. *Entrepreneurship: Theory and Practice*, *43*(1), 51–81. 10.1177/1042258718780431

[CR46] Cirillo, A., Ossorio, M., & Pennacchio, L. (2019). Family ownership and R&D investment: The moderating role of banks and private equity. *Management Decision,**57*(7), 1675–1694. 10.1108/MD-07-2016-0454

[CR47] Cobo, M. J., López-Herrera, A. G., Herrera-Viedma, E., & Herrera, F. (2011). An approach for detecting, quantifying, and visualizing the evolution of a research field: A practical application to the Fuzzy Sets Theory field. *Journal of Informetrics,**5*(1), 146–166. 10.1016/j.joi.2010.10.002

[CR48] Cohen, W. M., & Levinthal, D. A. (1990). Absorptive capacity: A new perspective on learning and innovation. *Administrative Science Quarterly,**35*(1), 128–152. 10.2307/2393553

[CR49] Cohn, J., Nestoriak, N., & Wardlaw, M. (2021). Private equity buyouts and workplace safety. *Review of Financial Studies,**34*(10), 4832–4875. 10.1093/rfs/hhab001

[CR50] Collewaert, V., Filatotchev, I., & Khoury, T. A. (2021). The view of angels from above: angel governance and institutional environments. *Academy of Management Perspectives, 35*(1), 9–24. 10.5465/amp.2017.0191

[CR51] Collings, D. G., Nyberg, A. J., Wright, P. M., & McMackin, J. (2021). Leading through paradox in a COVID-19 world: Human resources comes of age. *Human Resource Management Journal,**31*(4), 819–833. 10.1111/1748-8583.12343

[CR52] Colombo, M. G., Cumming, D. J., & Vismara, S. (2016). Governmental venture capital for innovative young firms. *Journal of Technology Transfer,**41*(1), 10–24. 10.1007/s10961-014-9380-9

[CR53] Colombo, M. G., D’Adda, D., & Quas, A. (2019). The geography of venture capital and entrepreneurial ventures’ demand for external equity. *Research Policy,**48*(5), 1150–1170. 10.1016/j.respol.2018.12.004

[CR54] Conti, A., Dass, N., Di Lorenzo, F., & Graham, S. J. H. (2019). Venture capital investment strategies under financing constraints: Evidence from the 2008 financial crisis. *Research Policy,**48*(3), 799–812. 10.1016/j.respol.2018.11.009

[CR55] Crane, D. (1969). Social Structure in a Group of Scientists : a Test of the “Invisible College”. *American Sociological Review*, *34*(3), 335–352. 1 10.2307/2092499

[CR56] Culnan, M. J. (1987). Mapping the intellectual structure of MIS, 1980–1985: A co-citation analysis. *MIS Quarterly: Management Information Systems,**11*(3), 341–350. 10.2307/248680

[CR57] Culnan, M. J., O’Reilly, C. A., III., & Chatman, J. A. (1990). Intellectual structure of research in organizational behavior, 1972–1984: A cocitation analysis. *Journal of the American Society for Information Science,**41*(6), 453–458. 10.1002/(SICI)1097-4571(199009)41:6%3c453::AID-ASI13%3e3.0.CO;2-E

[CR58] Cumming, D., & Walz, U. (2010). Private equity returns and disclosure around the world. *Journal of International Business Studies,**41*(4), 727–754. 10.1057/jibs.2009.62

[CR59] Brown, R., Rocha, A., & Cowling, M. (2020). Financing entrepreneurship in times of crisis: Exploring the impact of COVID-19 on the market for entrepreneurial finance in the United Kingdom. *International Small Business Journal-Researching Entrepreneurship, 38*(5), 380–390. 10.1177/0266242620937464 10.1177/0266242620937464 PMC7342936 38602995

[CR60] Cumming, D., & Zhang, M. (2019). Angel investors around the world. *Journal of International Business Studies,**50*(5), 692–719. 10.1057/s41267-018-0178-0

[CR61] Cumming, D., Siegel, D. S., & Wright, M. (2007). Private equity, leveraged buyouts and governance. *Journal of Corporate Finance,**13*(4), 439–460. 10.1016/j.jcorpfin.2007.04.008

[CR62] Cumming, D., Fleming, G., & Schwienbacher, A. (2009). Corporate relocation in venture capital finance. *Entrepreneurship Theory and Practice, 33*(5), 1121–1155. 10.1111/j.1540-6520.2009.00337.x

[CR63] Cumming, D. J., Johan, S. A., & Zhang, Y. (2019). The role of due diligence in crowdfunding platforms. *Journal of Banking and Finance,**108*, 105661. 10.1016/j.jbankfin.2019.105661

[CR64] Cumming, D., Meoli, M., & Vismara, S. (2019). Investors’ choices between cash and voting rights: Evidence from dual-class equity crowdfunding. *Research Policy,**48*(8), 103740. 10.1016/j.respol.2019.01.014

[CR65] Cumming, D., Meoli, M., & Vismara, S. (2021). Does equity crowdfunding democratize entrepreneurial finance? *Small Business Economics,**56*(2), 533–552. 10.1007/s11187-019-00188-z

[CR66] Cumming, D. J., & Johan, S. A. (2013). *Venture capital and private equity contracting *(2nd ed). Cambridge, Massachusetts: Elsevier Science Academic Press.

[CR67] Koenig, L., & Burghof, H.-P. (2022). The investment style drift puzzle and risk-taking in venture capital, Review of Corporate. *Finance, 2*, forthcoming.

[CR68] Cumming, D., Werth, J. C., & Zhang, Y. (2019c). *Governance in entrepreneurial ecosystems: venture capitalists vs. technology parks*. *Small Business Economics,**52*. 10.1007/s11187-017-9955-6

[CR69] Cumming, D. J., Martinez-Salgueiro, A., Reardon, R., & Sewaid, A. (2021a). COVID-19 bust, policy response, and rebound: Equity crowdfunding and P2P vs. Banks. *Journal of Technology Transfer*. 10.1007/s10961-021-09899-6. 10.1007/s10961-021-09899-6 PMC8520110 34690426

[CR70] Cummings, M. E., Rawhouser, H., & Vismara, S. (2020). An equity crowdfunding research agenda: evidence from stakeholder participation in the rulemaking process. *Small Business Economics, 54*(4), 907–932. 10.1007/s11187-018-00134-5

[CR71] Dai, N. (2022). Empirical research on private equity funds : A review of the past decade and future research opportunities. *Review of Corporate Finance*, *Forthcomin*(Forthcoming).

[CR72] de Leeuw, T., Gilsing, V., & Duysters, G. (2019). Greater adaptivity or greater control? Adaptation of IOR portfolios in response to technological change. *Research Policy,**48*(6), 1586–1600. 10.1016/j.respol.2018.12.003

[CR73] Delfim, J. C., & Hoesli, M. (2019). Real estate in mixed-asset portfolios for various investment horizons. *Journal of Portfolio Management,**45*(7), 141–158. 10.3905/jpm.2019.45.7.141

[CR74] Demirel, P., & Danisman, G. O. (2019). Eco-innovation and firm growth in the circular economy: Evidence from European small- and medium-sized enterprises. *Business Strategy and the Environment,**28*(8), 1608–1618. 10.1002/bse.2336

[CR75] Demiroglu, C., & James, C. M. (2010). The role of private equity group reputation in LBO financing. *Journal of Financial Economics,**96*(2), 306–330. 10.1016/j.jfineco.2010.02.001

[CR76] Ding, Y., Yan, E., Frazho, A., & Caverlee, J. (2009). PageRank for ranking authors in co-citation networks. *Journal of the American Society for Information Science,**60*(11), 2229–2243. 10.1002/asi

[CR77] Donthu, N., Kumar, S., Mukherjee, D., Pandey, N., & Lim, W. M. (2021). How to conduct a bibliometric analysis: An overview and guidelines. *Journal of Business Research,**133*(March), 285–296. 10.1016/j.jbusres.2021.04.070

[CR78] Dushnitsky, G., & Lavie, D. (2010). How alliance formation shapes corporate venture capital investment in the software industry: A resource-based perspective. *Strategic Entrepreneurship Journal,**4*(1), 22–48. 10.1002/sej.81

[CR79] Dushnitsky, G., & Lenox, M. J. (2005). When do firms undertake R&D by investing in new ventures? *Strategic Management Journal,**26*(10), 947–965. 10.1002/smj.488

[CR80] Dushnitsky, G., & Lenox, M. J. (2005). When do incumbents learn from entrepreneurial ventures?: Corporate venture capital and investing firm innovation rates. *Research Policy,**34*(5), 615–639. 10.1016/j.respol.2005.01.017

[CR81] Dushnitsky, G., & Shaver, J. M. (2009). Limitations to interorganizational knowledge acquisition: The paradox of corporate venture capital. *Strategic Management Journal,**30*(10), 1045–1064. 10.1002/smj.781

[CR82] Eaton, C., Howell, S. T., & Yannelis, C. (2020). When investor incentives and consumer interests diverge: Private equity in higher education. *Review of Financial Studies,**33*(9), 4024–4060. 10.1093/rfs/hhz129

[CR83] Ewens, M., & Farre-Mensa, J. (2020). The deregulation of the private equity markets and the decline in IPOs. *Review of Financial Studies,**33*(12), 5463–5509. 10.1093/rfs/hhaa053

[CR84] Fisch, C., & Momtaz, P. P. (2020). Institutional investors and post-ICO performance: an empirical analysis of investor returns in initial coin offerings (ICOs). *Journal of Corporate Finance*, *64*(July 2019), 101679. 10.1016/j.jcorpfin.2020.101679

[CR85] Fuchs, F., Füss, R., Jenkinson, T., & Morkoetter, S. (2021). Winning a deal in private equity: Do educational ties matter? *Journal of Corporate Finance*, *66*(July 2018), 101740. 10.1016/j.jcorpfin.2020.101740

[CR86] Fuchs, F., Füss, R., Jenkinson, T., & Morkoetter, S. (2022). Should investors care where private equity managers went to school? *Review of Corporate Finance* (Forthcoming)

[CR87] Gentry, M., & Stroup, C. (2019). Entry and competition in takeover auctions. *Journal of Financial Economics,**132*(2), 298–324. 10.1016/j.jfineco.2018.10.007

[CR88] Giraudo, E., Giudici, G., & Grilli, L. (2019). Entrepreneurship policy and the financing of young innovative companies: Evidence from the Italian Startup Act. *Research Policy,**48*(9), 103801. 10.1016/j.respol.2019.05.010

[CR89] Gompers, P. A. (1995). Optimal investment, monitoring, and the staging of venture capital. *The Journal of Finance,**50*(5), 1461–1489. 10.1111/j.1540-6261.1995.tb05185.x

[CR90] Gompers, P. A. (1996). Grandstanding in the venture capital industry. *Journal of Financial Economics,**42*(1), 133–156. 10.1016/0304-405X(96)00874-4

[CR91] Gompers, P. A., & Lerner, J. (1999). *The Venture Capital Cycle*. MIT Press.

[CR92] Gompers, P., & Lerner, J. (2000). Money chasing deals? The impact of fund inflows on private equity valuations. *Journal of Financial Economics,**55*(2), 281–325. 10.1016/S0304-405X(99)00052-5

[CR93] Gomulya, D., Jin, K., Lee, P. M., & Pollock, T. G. (2019). Crossed wires: Endorsement signals and the effects of IPO firm delistings on venture capitalists’ reputations. *Academy of Management Journal,**62*(3), 641–666. 10.5465/amj.2016.0796

[CR94] Gorman, M., & Sahlman, W. A. (1989). What do venture capitalists do? *Journal of Business Venturing,**4*(4), 231–248. 10.1016/0883-9026(89)90014-1

[CR95] Gornall, W., & Strebulaev, I. A. (2020). Squaring venture capital valuations with reality. *Journal of Financial Economics,**135*(1), 120–143. 10.1016/j.jfineco.2018.04.015

[CR96] Gou, X., Liao, H., Wang, X., Xu, Z., & Herrera, F. (2019). Consensus based on multiplicative consistent double hierarchy linguistic preferences: Venture capital in real estate market. *International Journal of Strategic Property Management,**24*(1), 1–23. 10.3846/ijspm.2019.10431

[CR97] Guler, I. (2007). Throwing good money after bad? political and institutional influences on sequential decision making in the venture capital industry. *Administrative Science Quarterly,**52*(2), 248–285. 10.2189/asqu.52.2.248

[CR98] Guo, B., Pérez-Castrillo, D., & Toldrà-Simats, A. (2019). Firms’ innovation strategy under the shadow of analyst coverage. *Journal of Financial Economics,**131*(2), 456–483. 10.1016/j.jfineco.2018.08.005

[CR99] Gupta, A. K., & Sapienza, H. J. (1992). Determinants of venture capital firms’ preferences regarding the industry diversity and geographic scope of their investments. *Journal of Business Venturing,**7*(5), 347–362. 10.1016/0883-9026(92)90012-G

[CR100] Guzman, J., & Kacperczyk, A. (Olenka). (2019). Gender gap in entrepreneurship. *Research Policy*, *48*(7), 1666–1680.10.1016/j.respol.2019.03.012

[CR101] Haddad, C., & Hornuf, L. (2019). The emergence of the global fintech market: Economic and technological determinants. *Small Business Economics,**53*(1), 81–105. 10.1007/s11187-018-9991-x

[CR102] Harris, R., Siegel, D. S., & Wright, M. (2005). Assessing the impact of management buyouts on economic efficiency: Plant-level evidence from the United Kingdom. *Review of Economics and Statistics,**87*(1), 148–153. 10.1162/0034653053327540

[CR103] Hellmann, T., & Puri, M. (2002). Venture capital and the professionalization of start-up firms: Empirical evidence. *Journal of Finance,**57*(1), 169–197. 10.1111/1540-6261.00419

[CR104] Ho, Y.-P., & Wong, P.-K. (2007). Financing, Regulatory costs and entrepreneurial propensity. *Small Business Economics,**28*(2–3), 187–204. 10.1007/s11187-006-9015-0

[CR105] Hochberg, Y. V., Ljungqvist, A., & Lu, Y. (2007). Whom you know matters: Venture capital networks and investment performance. *The Journal of Finance,**62*(1), 251–301. 10.1111/j.1540-6261.2007.01207.x

[CR106] Hota, P. K., Subramanian, B., & Narayanamurthy, G. (2019). Mapping the intellectual structure of social entrepreneurship research: A citation/co-citation analysis. *Journal of Business Ethics*, 1–26.10.1007/s10551-019-04129-4

[CR107] Howell, S. T., Niessner, M., & Yermack, D. (2020). Initial coin offerings: Financing growth with cryptocurrency token sales. *Review of Financial Studies,**33*(9), 3925–3974. 10.1093/rfs/hhz131

[CR108] Hsu, D. H. (2004). What do entrepreneurs pay for venture capital affiliation? *The Journal of Finance,**59*(4), 1805–1844. 10.1111/j.1540-6261.2004.00680.x

[CR109] Hsu, D. H., & Kenney, M. (2005). Organizing venture capital: the rise and demise of American Research & Development Corporation, 1946–1973. *Industrial and Corporate Change, 14*(4), 579–616. 10.1093/icc/dth064

[CR110] Huang, S. S., & Bowblis, J. R. (2019). Private equity ownership and nursing home quality: An instrumental variables approach. *International Journal of Health Economics and Management,**19*(3–4), 273–299. 10.1007/s10754-018-9254-z 30357589 10.1007/s10754-018-9254-z

[CR111] Huang, W., Meoli, M., & Vismara, S. (2020). The geography of initial coin offerings. *Small Business Economics,**55*(1), 77–102. 10.1007/s11187-019-00135-y

[CR112] Jelic, R., Zhou, D., & Wright, M. (2019). Sustaining the Buyout Governance Model: Inside Secondary Management Buyout Boards. *British Journal of Management,**30*(1), 30–52. 10.1111/1467-8551.12301

[CR113] Jensen, M. C. (1986). Agency Costs of Free Cash Flow, Corporate Finance, and Takeovers. *American Economic Review,**76*(2), 323–329.

[CR114] Johan, S., & Zhang, Y. (2021). Information asymmetries in private equity: Reporting frequency, endowments, and governance. *Journal of Business Ethics,**174*(1), 199–220.

[CR115] Levasseur, L., Johan, S., & Eckhardt, J. (2022). Mixed methods in venture capital research: an illustrative study and directions for future work. *British Journal of Management, 33*(1), 26–45. 10.1111/1467-8551.12514

[CR116] Johan, S., & Zhang, Y. (2021). Investors’ industry preference in equity crowdfunding. *Journal of Technology Transfer*. 10.1007/s10961-021-09897-8

[CR117] Kaplan, S. (1989). The effects of management buyouts on operating performance and value. *Journal of Financial Economics,**24*(2), 217–254. 10.1016/0304-405X(89)90047-0

[CR118] Kaplan, S. N., & Schoar, A. (2005). Private equity performance: Returns, persistence, and capital flows. *Journal of Finance,**60*(4), 1791–1823. 10.1111/j.1540-6261.2005.00780.x

[CR119] Kaplan, S. N., & Stromberg, P. (2003). Financial contracting theory meets the real world: An empirical analysis of venture capital contracts. *Review of Economic Studies,**70*(2), 281–315. 10.1111/1467-937X.00245

[CR120] Kaplan, S. N., & Strömberg, P. (2009). Leveraged buyouts and private equity. *Journal of Economic Perspectives,**23*(1), 121–146. 10.1257/jep.23.1.121

[CR121] Kim, J. Y. (Rose), Steensma, H. K., & Park, H. D. (2019). The influence of technological links, social ties, and incumbent firm opportunistic propensity on the formation of corporate venture capital deals. *Journal of Management*, *45*(4), 1595–1622.10.1177/0149206317720722

[CR122] Kortum, S., & Lerner, J. (2000). Assessing the contribution of venture capital to innovation. *The RAND Journal of Economics,**31*(4), 674–692.

[CR123] Lee, P. M., & Wahal, S. (2004). Grandstanding, certification and the underpricing of venture capital backed IPOs. *Journal of Financial Economics,**73*(2), 375–407. 10.1016/j.jfineco.2003.09.003

[CR124] Lee, C., Lee, K., & Pennings, J. M. (2001). Internal capabilities, external networks, and performance: A study on technology-based ventures. *Strategic Management Journal,**22*(6–7), 615–640. 10.1002/smj.181

[CR125] Leland, H. E., & Pyle, D. H. (1977). Informational Asymmetries, financial structure, and financial intermediation. *The Journal of Finance,**32*(2), 371. 10.2307/2326770

[CR126] Lerner, J. (1994). Venture capitalists to go public. *Journal of Financial Economics,**35*, 293–316.

[CR127] Lerner, J. (1995). Venture capitalists and the oversight of private firms. *The Journal of Finance,**50*(1), 301–318. 10.1111/j.1540-6261.1995.tb05175.x

[CR128] Lerner, J., & Nanda, R. (2020). Venture capital’s role in financing innovation: What we know and how much we still need to learn. *Journal of Economic Perspectives,**34*(3), 237–261. 10.1257/jep.34.3.237

[CR129] Lerner, J., Sorensen, M., & Strömberg, P. (2011). Private equity and long-run investment: The case of innovation. *The Journal of Finance, 66*(2), 445–477. 10.1111/j.1540-6261.2010.01639.x

[CR130] Lichtenberg, F. R., & Siegel, D. (1990). The effects of leveraged buyouts on productivity and related aspects of firm behavior. *Journal of Financial Economics,**27*(1), 165–194. 10.1016/0304-405X(90)90025-U

[CR131] Liu, X., Wang, Z., Zhang, S., & Liu, J. (2020). Probabilistic hesitant fuzzy multiple attribute decision-making based on regret theory for the evaluation of venture capital projects. *Economic Research-Ekonomska Istrazivanja,**33*(1), 672–697. 10.1080/1331677X.2019.1697327

[CR132] Luo, J. D., Rong, K., Yang, K., Guo, R., & Zou, Y. Q. (2019). Syndication through social embeddedness: A comparison of foreign, private and state-owned venture capital (VC) firms. *Asia Pacific Journal of Management,**36*(2), 499–527. 10.1007/s10490-017-9561-9

[CR133] Ma, S. (2020). The Life Cycle of Corporate Venture Capital. *The Review of Financial Studies,**33*(1), 358–394. 10.1093/rfs/hhz042

[CR134] MacCoun, R. J. (1998). Biases in the interpretation and use of research results. *Annual Review of Psychology,**49*(1), 259–287. 10.1146/annurev.psych.49.1.259 10.1146/annurev.psych.49.1.259 15012470

[CR135] Macmillan, I. C., Siegel, R., & Narasimha, P. N. S. (1985). Criteria used by venture capitalists to evaluate new venture proposals. *Journal of Business Venturing,**1*(1), 119–128. 10.1016/0883-9026(85)90011-4

[CR136] Macmillan, I. C., Kulow, D. M., & Khoylian, R. (1989). Venture capitalists’ involvement in their investments: Extent and performance. *Journal of Business Venturing,**4*(1), 27–47. 10.1016/0883-9026(89)90032-3

[CR137] Megginson, W. L., & Weiss, K. A. (1991). Venture Capitalist Certification in Initial Public Offerings. *The Journal of Finance,**46*(3), 879–903. 10.1111/j.1540-6261.1991.tb03770.x

[CR138] Megginson, W. L., Meles, A., Sampagnaro, G., & Verdoliva, V. (2019). Financial distress risk in initial public offerings: How much do venture capitalists matter? *Journal of Corporate Finance,**59*, 10–30. 10.1016/j.jcorpfin.2016.09.007

[CR139] Meoli, M., & Vismara, S. (2022). Machine-Learning Forecasting of Successful ICOs, Journal of Economics and Business, 106071.10.1016/j.jeconbus.2022.106071

[CR140] Metrick, A., & Yasuda, A. (2010). The economics of private equity funds. *Review of Financial Studies,**23*(6), 2303–2341. 10.1093/rfs/hhq020

[CR141] Morris, P., & Phalippou, L. (2020). Thirty years after Jensen’s prediction: Is private equity a superior form of ownership? *Oxford Review of Economic Policy,**36*(2), 291–313. 10.1093/oxrep/graa004

[CR142] Moskowitz, T. J., & Vissing-Jørgensen, A. (2002). The returns to entrepreneurial investment: A private equity premium puzzle? *American Economic Review,**92*(4), 745–778. 10.1257/00028280260344452

[CR143] Mukherjee, D., Lim, W. M., Kumar, S., & Donthu, N. (2022). Guidelines for advancing theory and practice through bibliometric research. *Journal of Business Research, 148*, 101–115.

[CR144] Myers, S. C., & Majluf, N. S. (1984). Corporate financing and investment decisions when firms have information that investors do not have. *Journal of Financial Economics,**13*(1), 187–221.

[CR145] Nadauld, T. D., Sensoy, B. A., Vorkink, K., & Weisbach, M. S. (2019). The liquidity cost of private equity investments: Evidence from secondary market transactions. *Journal of Financial Economics,**132*(3), 158–181. 10.1016/j.jfineco.2018.11.007

[CR146] Nahata, R. (2008). Venture capital reputation and investment performance. *Journal of Financial Economics,**90*(2), 127–151. 10.1016/j.jfineco.2007.11.008

[CR147] Nazareno, J., Zhou, M., & You, T. (2019). Global dynamics of immigrant entrepreneurship: Changing trends, ethnonational variations, and reconceptualizations. *International Journal of Entrepreneurial Behaviour and Research,**25*(5), 780–800. 10.1108/IJEBR-03-2018-0141

[CR148] Nerur, S. P., Rasheed, A. A., & Natarajan, V. (2008). The intellectual structure of the strategic management field: An author co-citation analysis. *Strategic Management Journal,**29*(3), 319–336. 10.1002/smj.659

[CR149] Norton, E., & Tenenbaum, B. H. (1993). Specialization versus diversification as a venture capital investment strategy. *Journal of Business Venturing,**8*(5), 431–442. 10.1016/0883-9026(93)90023-X

[CR150] Opler, T., & Titman, S. (1993). American finance association the determinants of leveraged buyout activity : Free cash flow vs . financial distress costs author ( s ): Tim Opler and Sheridan Titman Source : The Journal of Finance , Vol . 48 , No . 5 ( Dec ., 1993 ), pp . 1985–1999 Publi. *Journal of Finance*, *48*(5), 1985–1999.

[CR151] Ozmel, U., Trombley, T. E., & Yavuz, M. D. (2019). Outside insiders: Does access to information prior to an ipo generate a trading advantage after the IPO? *Journal of Financial and Quantitative Analysis,**54*(1), 303–334. 10.1017/S0022109018000546

[CR152] Pan, F., & Yang, B. (2019). Financial development and the geographies of startup cities: Evidence from China. *Small Business Economics,**52*(3), 743–758. 10.1007/s11187-017-9983-2

[CR153] Pan, L., Li, X., Chen, J., & Chen, T. (2019). Sounds novel or familiar? Entrepreneurs’ Framing Strategy in the Venture Capital Market. *Journal of Business Venturing*. 10.1016/j.jbusvent.2019.02.003

[CR154] Phalippou, L., & Gottschalg, O. (2009). The performance of private equity funds. *Review of Financial Studies,**22*(4), 1747–1776. 10.1093/rfs/hhn014

[CR155] Pittaway, L., Robertson, M., Munir, K., Denyer, D., & Neely, A. (2004). Networking and innovation: A systematic review of the evidence. *International Journal of Management Reviews,**5–6*(3–4), 137–168. 10.1111/j.1460-8545.2004.00101.x

[CR156] Platanakis, E., Sakkas, A., & Sutcliffe, C. (2019). Harmful diversification: Evidence from alternative investments. *British Accounting Review,**51*(1), 1–23. 10.1016/j.bar.2018.08.003

[CR157] Quas, A., Martí, J., & Reverte, C. (2021). What money cannot buy: A new approach to measure venture capital ability to add non-financial resources. *Small Business Economics,**57*(3), 1361–1382. 10.1007/s11187-020-00352-w

[CR158] Que, J., & Zhang, X. (2020). The role of foreign and domestic venture capital in innovation: Evidence from China. *Accounting and Finance,**60*(S1), 1077–1110. 10.1111/acfi.12401

[CR159] Ramos-Rodrígue, A. R., & Ruíz-Navarro, J. (2004). Changes in the intellectual structure of strategic management research: A bibliometric study of the Strategic Management Journal, 1980–2000. *Strategic Management Journal,**25*(10), 981–1004. 10.1002/smj.397

[CR160] Revest, V., & Sapio, A. (2012). Financing technology-based small firms in Europe: What do we know? *Small Business Economics*. 10.1007/s11187-010-9291-6

[CR161] Robinson, M. (2022). Factors impacting entrepreneurial success in accelerators: revealed preferences of sophisticated mentors. *Review of Corporate Finance, 2*, forthcoming.

[CR162] Rossi, M., Festa, G., Devalle, A., & Mueller, J. (2020). When corporations get disruptive, the disruptive get corporate: Financing disruptive technologies through corporate venture capital. *Journal of Business Research,**118*(February), 378–388. 10.1016/j.jbusres.2020.07.004

[CR163] Sahlman, W. A. (1990). The structure and governance of venture-capital organizations. *Journal of Financial Economics,**27*(2), 473–521. 10.1016/0304-405X(90)90065-8

[CR164] Sakawa, H., & Watanabel, N. (2020). IPO underpricing and ownership monitoring in Japan. *Asian Business and Management,**19*(4), 480–503. 10.1057/s41291-019-00067-1

[CR165] Samiee, S., & Chabowski, B. R. (2012). Knowledge structure in international marketing: A multi-method bibliometric analysis. *Journal of the Academy of Marketing Science,**40*(2), 364–386. 10.1007/s11747-011-0296-8

[CR166] Sapienza, H. J. (1992). When do venture capitalists add value? *Journal of Business Venturing,**7*(1), 9–27. 10.1016/0883-9026(92)90032-M

[CR167] Sapienza, H. J., Manigart, S., & Vermeir, W. (1996). Venture capitalist governance and value added in four countries. *Journal of Business Venturing,**11*(6), 439–469. 10.1016/S0883-9026(96)00052-3

[CR168] Signori, A., & Vismara, S. (2018). Does success bring success? The post-offering lives of equity-crowdfunded firms. *Journal of Corporate Finance,**50*, 575–591. 10.1016/j.jcorpfin.2017.10.018

[CR169] Small, H. (1973). Co-citation in the scientific literature : A new measure of the relationship between two documents. *Journal of the American Society for Information Science,**24*(4), 265–269. 10.1002/asi.4630240406

[CR170] Smith, E. E., Smith, J. K., & Smith, R. L. (2022). Bias in the reporting of venture capital performance: The disciplinary role of FOIA. *Review of Corporate Finance* (Forthcoming).

[CR171] Sorenson, O., & Stuart, T. E. (2001). Syndication networks and the spatial distribution of venture capital investments. *American Journal of Sociology,**10*(6), 1546–1588. 10.1086/321301

[CR172] Stuart, T. E., Hoang, H., & Hybels, R. C. (1999). Interorganizational endorsements and the performance of entrepreneurial ventures. *Administrative Science Quarterly,**44*(2), 315–349. 10.2307/2666998

[CR173] Sun, S. L., Chen, V. Z., Sunny, S. A., & Chen, J. (2019). Venture capital as an innovation ecosystem engineer in an emerging market. *International Business Review*, *28*(5), 0–1.10.1016/j.ibusrev.2018.02.012

[CR174] Tian, X., Kou, G., & Zhang, W. (2020). Geographic distance, venture capital and technological performance: Evidence from Chinese enterprises. *Technological Forecasting and Social Change,**158*(June), 120155. 10.1016/j.techfore.2020.120155

[CR175] Tranfield, D., Denyer, D., & Smart, P. (2003). Towards a methodology for developing evidence-informed management knowledge by means of systematic review. *British Journal Management,**14*(3), 207–222. 10.1111/1467-8551.00375

[CR176] Tyebjee, T. T., & Bruno, A. V. (1984). Model of venture capitalist investment activity. *Management Science,**30*(9), 1051–1066. 10.1287/mnsc.30.9.1051

[CR177] van Eck, N. J., & Waltman, L. (2010). Software survey: VOSviewer, a computer program for bibliometric mapping. *Scientometrics,**84*(2), 523–538. 10.1007/s11192-009-0146-3 20585380 10.1007/s11192-009-0146-3 PMC2883932

[CR178] Vanacker, T., Forbes, D. P., Knockaert, M., & Manigart, S. (2020). Signal strength, media attention, and resource mobilization: Evidence from new private equity firm. *Academy of Management Journal,**63*(4), 1082–1105. 10.5465/AMJ.2018.0356

[CR179] Vismara, S. (2016). Equity retention and social network theory in equity crowdfunding. *Small Business Economics,**46*(4), 579–590. 10.1007/s11187-016-9710-4

[CR180] Vismara, S. (2019). Sustainability in equity crowdfunding. *Technological Forecasting and Social Change,**141*(May), 98–106. 10.1016/j.techfore.2018.07.014

[CR181] Vismara, S. (2022). Expanding corporate finance perspectives to equity crowdfunding. *Journal of Technology Transfer, Forthcoming*. 10.1007/s10961-021-09903-z 10.1007/s10961-021-09903-z PMC8591156 34803218

[CR182] Wadhwa, A., & Kotha, S. (2006). Knowledge creation through external venturing: Evidence from the telecommunications equipment manufacturing industry. *Academy of Management Journal,**49*(4), 819–835. 10.5465/amj.2006.22083132

[CR183] Weinberg, B. H. (1974). Bibliographic coupling: A review. *Information Storage and Retrieval,**10*(5–6), 189–196. 10.1016/0020-0271(74)90058-8

[CR184] Wright, M., & Lockett, A. (2003). The structure and management of alliances: Syndication in the venture capital industry. *Journal of Management Studies,**40*(8), 2073–2102. 10.1046/j.1467-6486.2003.00412.x

[CR185] Wright, M., Hoskisson, R. E., Busenitz, L. W., & Dial, J. (2000). Entrepreneurial growth through privatization: The upside of management buyouts. *Academy of Management Review,**25*(3), 591–601. 10.5465/AMR.2000.3363522

[CR186] Wu, L., & Xu, L. (2020). Venture capital certification of small and medium-sized enterprises towards banks: Evidence from China. *Accounting and Finance,**60*(2), 1601–1633. 10.1111/acfi.12489

[CR187] Xu, X., Gong, Y., Jia, F., Brown, S., & Xu, Y. (2018). Supply chain finance: A systematic literature review and bibliometric analysis. *International Journal of Production Economics,**204*, 160–173. 10.1016/j.ijpe.2018.08.003

[CR188] Yung, C. (2019). Entrepreneurial manipulation with staged financing. *Journal of Banking and Finance,**100*, 273–282. 10.1016/j.jbankfin.2018.06.016

[CR189] Zhang, W., Tian, X., & Yu, A. (2020). Is high-speed rail a catalyst for the fourth industrial revolution in China? Story of enhanced technology spillovers from venture capital. *Technological Forecasting and Social Change*, *161*(August). 10.1016/j.techfore.2020.120286

